# Electrochemical Synthesis of Disubstituted Alkynes from Dihydrazones

**DOI:** 10.1021/acs.orglett.5c01968

**Published:** 2025-06-23

**Authors:** Subhabrata Dutta, Jacob Kayser, Siegfried R. Waldvogel

**Affiliations:** † Department of Electrosynthesis, Max-Planck-Institute for Chemical Energy Conversion, Stiftstraße 34−36, 45479 Mulheim an der Ruhr, Germany; ‡ Department of Chemistry, 9182 Johannes Gutenberg-University Mainz, Duesbergweg 10−14, 55128 Mainz, Germany; § Institute of Biological and Chemical Systems−Functional Molecular Systems (IBCS−FMS), Karlsruhe Institute of Technology, Kaiserstraße 12, 76131 Karlsruhe, Germany

## Abstract

A simple, straightforward, scalable, and sustainable electrochemical transformation of dihydrazones to alkynes via anodic oxidation has been developed. The protocol operates under galvanostatic conditions in a commercially available undivided cell utilizing reusable carbon-based electrodes. This methodology enables the efficient synthesis of a broad range of alkynes with moderate to excellent yield. The successful demonstration of gram-scale synthesis, along with a consistent electrode reusability profile, underlines the synthetic utility and preparative potential of the approach.

## Graphical abstract

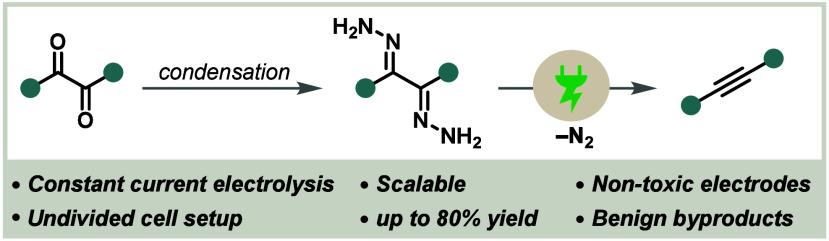

Alkynes, particularly disubstituted alkynes, are indispensable synthetic intermediates in modern organic chemistry with wide-ranging applications across pharmaceuticals, natural products, drug discovery, and materials science.^1,2^ Their linear geometry, structural rigidity, and chemical stability make them an ideal choice for linkers in the construction of conjugated systems and rigid rod-like polymers, imparting distinct physical and electronic properties to the molecular framework (Scheme 1A).^3–5^ In addition, their versatility as intermediates in cycloadditions,^6^ hydrogenation,^7,8^ click chemistry,^9^ metathesis,^10^ and cross-coupling^11–13^ reactions highlights their role in the development of diverse synthetic routes. Conventional methods for synthesizing disubstituted alkynes typically rely on metal-mediated cross-coupling reactions (Sonogashira^14,15^ or Cadiot–Chodkiewicz^16^ coupling) or halide-based β-elimination systems (Scheme 1B).^2^ In addition, some longer routes also involve starting from the carbonyl compound (Corey–Fuchs procedure) and converting them into dihalides.^17,18^ The ensuing step undergoes double elimination to offer the desired alkynes. However, these approaches often suffer from harsh reaction conditions, often using strong base, limited functional group tolerance, poor atom economy, and the generation of toxic byproducts and waste.^19,20^ While there are ample procedures available for terminal alkyne synthesis, the reported procedures often suffer from limitations, such as regioisomeric mixtures and the formation of undesired homocoupled byproducts. Such constraints demand to enroute an efficient and easy access toward alkynes.

**Scheme 1:**
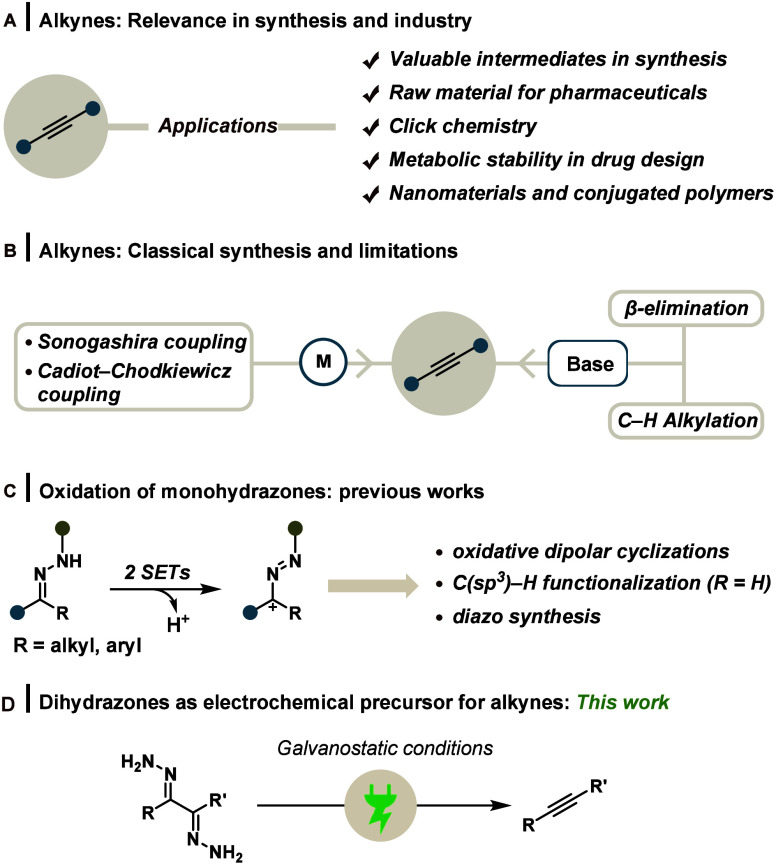
(A) Importance of Alkynes, (B) Methods for Synthesizing Alkynes and Their Shortcomings, (C) Previous Literature Reports Based on Anodic Oxidation of Hydrazones, and (D) This Work

Electrochemical synthesis provides a green, sustainable platform by using electricity, ideally from renewable sources, as a cost-effective and clean reagent.^21–26^ This field circumvents the overuse of stoichiometric external oxidants, paving the way for overall good reaction economy and better waste management.^27,28^ Keeping all this in mind, we set out to develop a method for synthesizing alkynes from a commercially available material, ensuring the ease of byproduct removal and improved process sustainability. Employing dihydrazones as a masked alkyne equivalent appears to be an ideal starting point for this approach (Scheme 1C).^29,30^ The redox property and the electro-oxidative transformations of hydrazone derivatives have been extensively studied in the literature. This includes the synthesis of diazo compounds,^31^ functionalized hydrazones,^32,33^ and varieties of aza-cycles via formal cycloaddition.^34–37^ By leveraging the oxidative cleavage of N–H bonds,^38,39^ followed by the cleavage of double C–N bonds and *in situ* N_2_ extrusion, we propose a streamlined electrochemical method for the formation of internal alkynes through double C–C bond formation under mild conditions (Scheme 1D). The generation of environmentally benign byproducts along with the potential for scalability makes this strategy particularly appealing for technical applications. Additionally, no external mediator is required to initiate the reaction.^40,41^

We commenced our investigations and optimizations using hydrazone **1a** as the alkyne equivalent. The compound was synthesized in a single-step condensation of the corresponding diketone and hydrazine hydrate with EtOH as the solvent under refluxing conditions (see the Supporting Information). Inspired by the study of the Lam group,^31^ we initiated the screen with graphite as the anode material and platinum as the anode. An initial combination of electrolytes, NH_4_OAc and KBr, yielded the target alkyne in 43%. Through a series of iterative optimizations, we ultimately achieved an improved yield of 81% (GC–FID) under ambient galvanostatic conditions. The corresponding counter reaction is the hydrogen evolution reaction (HER), which is compatible with the electrolytes and cost-efficient stainless-steel cathode.^42^

While the reaction appears to proceed via an oxidative mechanism, varying the anode material (Table 1, entries 2–4) did not lead to any further enhancement in the yield. Switching the solvent to a combination of MeCN/AcOH to ensure enough proton source also led to decreased reactivity (Table 1, entry 5). Interestingly, changing the supporting electrolyte to NBu_4_BF_4_ ceased the product formation completely (Table 1, entry 6). In addition, we also evaluated H_2_SO_4_ as an inexpensive proton source and electrolyte. However, similar to the previous observations, its use was detrimental to the reaction, with no product formation being detected (Table 1, entry 7). Furthermore, deviations in the current density (Table 1, entry 8) and amount of KOAc (Table 1, entry 9) also had a detrimental effect on the yield.

**Table 1:**
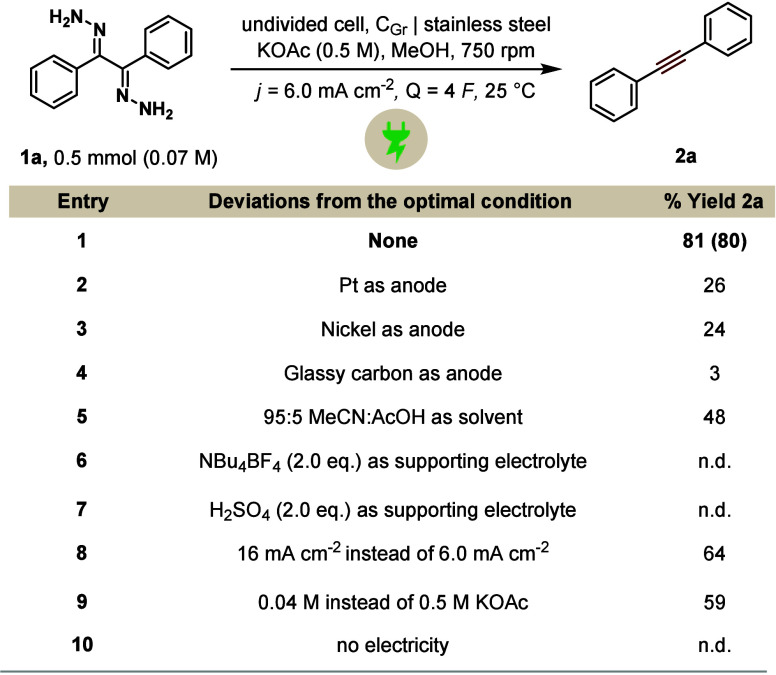
Deviation Studies^a^

a Reactions were performed on a 0.5 mmol scale. Yields were determined by GC–FID analysis using 1,3,5-trimethoxybenzene as the internal standard. Isolated yields are indicated in parentheses. n.d. = not detected. Further details about the experimental setup can be found in the Supporting Information.

To assess the reproducibility of the system, we investigated the influence of the temperature, stirring speed, and electrolyte concentration.^43^ Variations in these parameters, whether increased or decreased, did not significantly deviate from the optimized yield (see the Supporting Information). Notably, raising the reaction temperature to 50 °C maintained an efficiency comparable to that under the optimized conditions. Finally, the control experiment demonstrated that the reaction did not occur in the absence of electricity (entry 10). To elucidate the synergistic effects of electrochemical parameters, we initially employed a fractional factorial design of experiments (DoE) using a 2^5–2^ setup.^44–46^ The investigated variables included the electrolyte concentration, temperature, amount of applied charge, current density, and quantity of the starting material. Statistical analysis of the results identified the amount of applied charge as the most influential parameter governing the reaction outcome. Further evaluation revealed that consumption of a smaller amount of applied charge (*Q*), lower current density (*j*), and moderate temperature (*T*) were key factors in optimizing the product yield. In contrast, variations in the amount of starting material and the electrolyte concentration exhibited no significant impact. Based on the observed linear trends, we selected 25 °C, a low current density of 6.0 mA cm^–2^, and an applied charge of 4 *F* as the optimal conditions, which is in fact the theoretical amount of charge required for the presented protocol (see the Supporting Information for more details).

Having optimized the conditions, we set out to test the generality of the protocol (Scheme 2). We synthesized all dihydrazones from their corresponding 1,2-diketones using a simple condensation protocol with hydrazine. Testing different alkyl substituents on the aromatic ring offered desired diaryl alkynes **2a**–**2c** in moderate to excellent yields. Notably, fluoro (**2d**) and chloro (**2e**) were equally well-tolerated under the reaction conditions. Interestingly, an electron-rich substituent lowered the performance of the reaction. Several dihydrazones with differently substituted OMe groups (**2f**–**2h**) yielded the corresponding alkyne in moderate yields. Moving away from symmetrical systems, a combination of aryl and aliphatic side chains is also well-suited for the reaction, providing the phenyl acetylene derivatives **2i** and **2j** in good yields. Purely aliphatic alkynes could also be viable through our methodology, as shown in entry **2k**. Despite the presence of several functional groups, including ether and alkyne moieties, the target product was obtained in a 47% yield. While methoxy-substituted electron-rich systems were compatible, other electron-donating groups (**2l**–**2m**) did not withstand the reaction conditions.

**Scheme 2:**
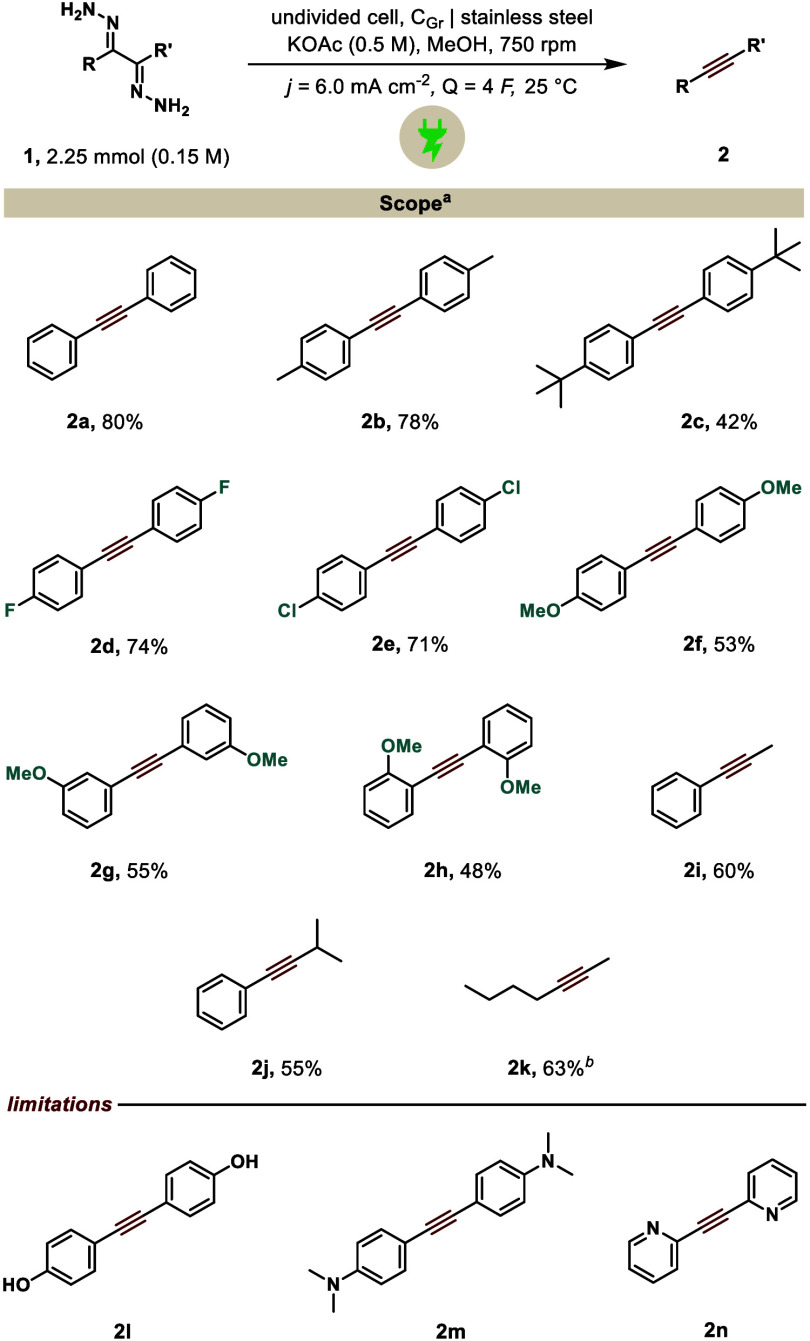
a Reaction conditions: compound **1** (2.25 mmol, 0.15 M), KOAc (0.5 M), MeOH, 6.0 mA cm^–2^, and 4 *F* under ambient conditions with 750 rpm as the stirring speed.
b The yield was analyzed using ^1^H NMR. Only decomposition was observed in substrates listed under “limitations”.

Additionally, pyridine-based hydrazone **2n** also failed under the optimized conditions. When performed on a doubled scale (1 mmol) compared to the optimized version (0.5 mmol), the transformation proceeded efficiently, affording the desired product **2a** in a 73% GC–FID yield (Scheme 3). Encouragingly, scaling up the reaction to 15 mmol, with a 6.7-fold increase and within the gram-scale range, also proved successful. From a sustainability perspective,^47^ we evaluated the reusability of the electrodes by conducting the reaction on a 1.0 mmol scale, utilizing electrodes with dimensions of 1 × 7 cm (Figure 1). The same set of electrodes was used over the course of 8 consecutive reaction cycles. Remarkably, only a modest decline in the yield was recorded, approximately 10% by the final run.

**Scheme 3:**
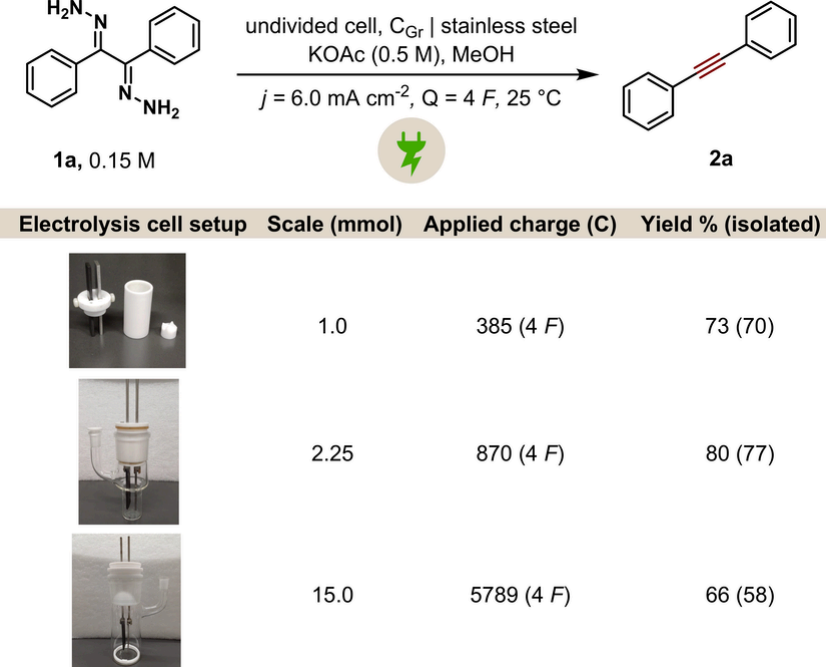
Scalability Test for the Synthesis of Product **2a**

**Figure 1:**
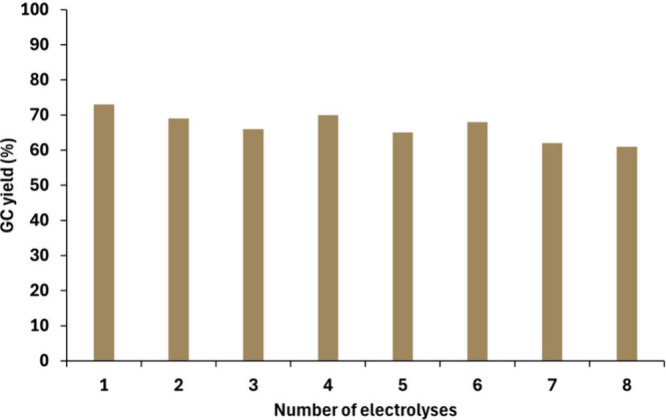
Reusability test for electrodes conducted with 8 consecutive electrolysis experiments.

Throughout these cycles, the reaction yields were in the range of 63–73%. This demonstrates that the electrodes can be effectively reused multiple times, supporting overall sustainability of the process.

With the synthetic features developed, we investigated the mechanistic route. Cyclic voltammetry experiments of compound **1a** reveal two distinct oxidation events (*E*_vs FcH/FcH^+^_ = 0.66 and 1.15 V), with the first being more prominent than the second. This dual oxidation pattern is consistent with the presence of two N–H bonds in the molecule. Based on established literature precedents regarding the oxidation of hydrazones,^34,48^ we propose the following reaction mechanism (Figure 2).

**Figure 2:**
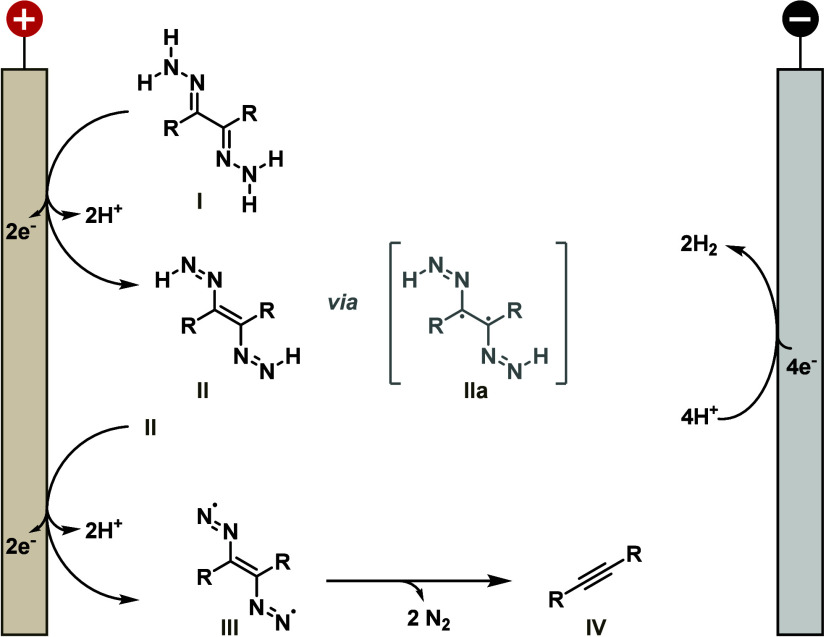
Proposed mechanism for the synthesis of alkyne **V**.

Following the first oxidation step of intermediate **I** with the loss of two protons, a diradical intermediate **IIa** is formed at the anodic surface. This gives rise to alkene-type intermediate **II**. The second N–H bond oxidation of intermediate **II** resulted in the formation of intermediate **III**. The ensuing steps comprise radical–radical recombination and N_2_ extrusion, affording desired alkyne **IV**. On the cathodic site, hydrogen gas is evolved as an efficient and non-intrusive counter reaction.

In summary, we established a straightforward, efficient, robust, scalable, and sustainable electrosynthetic method for the preparation of alkynes. The process begins with commercially available diketones, which undergo quantitative condensation with hydrazine to form dihydrazones, which are used as the key starting materials. The reaction was optimized using the DoE as a handy optimization tool. The reaction employs inexpensive KOAc as the supporting electrolyte. Moreover, the protocol uses a theoretical amount of charge, ensuring high atom and faradaic efficiency while minimizing energy consumption. The synthetic utility of the methodology was demonstrated through a broad substrate scope with up to 80% yield, showing good tolerance toward various halides and electron-donating groups and enabling access to a range of differently substituted aliphatic and aromatic systems. Furthermore, successful scalability and electrode reusability tests highlight the practical applicability and robustness of the protocol. A mechanistic blueprint is also proposed, accounting for double oxidation steps for hydrazones.

## Supplementary Material



## Data Availability

The data underlying this study are available in the published article and its Supporting Information.

## Notes

General information, screening and optimization, control experiments, experimental and characterization data, scalability test, reusability test, cyclic voltammetry studies, and spectra (PDF)

## References

[1] Bortolami M., Petrucci R., Rocco D., Scarano V., Chiarotto I. (2021). Alkynes as
Building Blocks, Intermediates and Products in the Electrochemical
Procedures Since 2000. ChemElectroChem..

[2] Shaw R., Elagamy A., Althagafi I., Pratap R. (2020). Synthesis of alkynes
from non-alkyne sources. Org. Biomol. Chem..

[3] Boeck P. T., Veige A. S. (2024). Cyclic polymers
from alkynes: a review. Chem. Sci..

[4] Boeck P. T., Yadav R., Sumerlin B. S., Veige A. S. (2024). Cyclic Polymers
from Alkynes: Scope and Degradation. Macromolecules.

[5] Liu Y., Lam J. W. Y., Tang B. Z. (2015). Conjugated
polymers developed from
alkynes. Natl. Sci. Rev..

[6] Meldal M., Tornøe C. W. (2008). Cu-catalyzed
azide-alkyne cycloaddition. Chem. Rev..

[7] Liu Z., Zhang L., Ren Z., Zhang J. (2023). Advances in Selective
Electrocatalytic Hydrogenation of Alkynes to Alkenes. Chem. - Eur. J..

[8] Sk M., Haldar S., Bera S., Banerjee D. (2024). Recent advances in
the selective semi-hydrogenation of alkyne to (E)-olefins. Chem. Commun..

[9] Kolb H. C., Finn M. G., Sharpless K. B. (2001). Click Chemistry: Diverse Chemical
Function from a Few Good Reactions. Angew. Chem.
Int. Ed..

[10] Fürstner A. (2013). Alkyne metathesis
on the rise. Angew. Chem. Int. Ed..

[11] Negishi E.-I., Wang G., Rao H., Xu Z. (2010). Alkyne elementometalation-Pd-catalyzed
cross-coupling. Toward synthesis of all conceivable types of acyclic
alkenes in high yields, efficiently, selectively, economically, and
safely: ″green″ way. J. Org. Chem..

[12] Wu W., Jiang H. (2014). Haloalkynes: a powerful
and versatile building block in organic synthesis. Acc. Chem. Res..

[13] Chinchilla R., Nájera C. (2014). Chemicals from alkynes with palladium catalysts. Chem. Rev..

[14] Karak M., Barbosa L. C. A., Hargaden G. C. (2014). Recent mechanistic developments and
next generation catalysts for the Sonogashira coupling reaction. RSC Adv..

[15] Chinchilla R., Najera C. (2007). The Sonogashira reaction: a booming methodology in
synthetic organic chemistry. Chem. Rev..

[16] Chinta B. S., Baire B. (2016). A systematic study
on the Cadiot–Chodkiewicz cross coupling
reaction for the selective and efficient synthesis of hetero-diynes. RSC Adv..

[17] Corey E. J., Fuchs P. L. (1972). A synthetic method for formyl→ethynyl conversion
(RCHO→RC≡CH or RC≡CR′). Tetrahedron Lett..

[18] Habrant D., Rauhala V., Koskinen A. M. P. (2010). Conversion of
carbonyl compounds
to alkynes: general overview and recent developments. Chem. Soc. Rev..

[19] Sahu B., Muruganantham R., Namboothiri I. N. N. (2007). Synthetic and Mechanistic Investigations
on the Rearrangement of 2,3-Unsaturated 1,4-Bis­(alkylidene)­carbenes
to Enediynes. Eur. J. Org. Chem..

[20] Campbell K. N., Campbell B. K. (1963). Org. Synth..

[21] Horn E. J., Rosen B. R., Baran P. S. (2016). Synthetic
Organic Electrochemistry:
An Enabling and Innately Sustainable Method. ACS Cent. Sci..

[22] Leech M. C., Garcia A. D., Petti A., Dobbs A. P., Lam K. (2020). Organic electrosynthesis:
from academia to industry. React. Chem. Eng..

[23] Pollok D., Waldvogel S. R. (2020). Electro-organic
synthesis—A 21st century technique. Chem.
Sci..

[24] Wang D., Li J., Li N., Gao T., Hou S., Chen B. (2010). An efficient
approach to homocoupling of terminal alkynes: Solvent-free synthesis
of 1,3-diynes using catalyticCu­(ii) and base. Green Chem..

[25] Möhle S., Zirbes M., Rodrigo E., Gieshoff T., Wiebe A., Waldvogel S. R. (2018). Modern
Electrochemical Aspects for the Synthesis of
Value-Added Organic Products. Angew. Chem. Int.
Ed..

[26] Wiebe A., Gieshoff T., Möhle S., Rodrigo E., Zirbes M., Waldvogel S. R. (2018). Electrifying Organic Synthesis. Angew. Chem. Int. Ed..

[27] Seidler J., Strugatchi J., Gärtner T., Waldvogel S. R. (2020). Does electrifying
organic synthesis pay off? The energy efficiency of electro-organic
conversions. MRS Energy Sustain..

[28] Waldvogel S. R., Janza B. (2014). Renaissance of electrosynthetic methods for the construction of complex
molecules. Angew. Chem. Int. Ed..

[29] Claraz A. (2024). Harnessing
the versatility of hydrazones through electrosynthetic oxidative transformations. Beilstein J. Org. Chem..

[30] Kong Y., Wei K., Yan G. (2022). Radical coupling reactions of hydrazines via photochemical
and electrochemical strategies. Org. Chem. Front..

[31] Tanbouza N., Petti A., Leech M. C., Caron L., Walsh J. M., Lam K., Ollevier T. (2022). Electrosynthesis
of Stabilized Diazo Compounds from
Hydrazones. Org. Lett..

[32] Wen J., Zhang L., Yang X., Niu C., Wang S., Wei W., Sun X., Yang J., Wang H. (2019). H 2 O-controlled selective
thiocyanation and alkenylation of ketene dithioacetals under electrochemical
oxidation. Green Chem..

[33] Xu Z., Li Y., Mo G., Zheng Y., Zeng S., Sun P.-H., Ruan Z. (2020). Electrochemical
Oxidative Phosphorylation of Aldehyde Hydrazones. Org. Lett..

[34] Zhang H., Ye Z., Chen N., Chen Z., Zhang F. (2022). Electrochemical dehydrogenative
C–N coupling of hydrazones for the synthesis of 1 H -indazoles. Green Chem..

[35] Linden M., Hofmann S., Herman A., Ehler N., Bär R. M., Waldvogel S. R. (2023). Electrochemical Synthesis of Pyrazolines and Pyrazoles
via 3 + 2 Dipolar Cycloaddition. Angew. Chem.,
Int. Ed..

[36] Yang M., Jiang R., Mu Y., Hong Y., Wan Y., Hou J., Tang D. (2023). Electrochemical cycloaddition of hydrazones with cyanamide
for the synthesis of substituted 5-amine-1,2,4-triazoles. Chem. Commun..

[37] Linden M., Hofmann S., Weber F. N., Bär R. M., Kaldas S. J., Ford M. J., Waldvogel S. R. (2023). From screening
to the hectogram scale: sustainable electrochemical synthesis of mefenpyr-diethyl. Green Chem..

[38] Gieshoff T., Kehl A., Schollmeyer D., Moeller K. D., Waldvogel S. R. (2017). Insights
into the Mechanism of Anodic N-N Bond Formation by Dehydrogenative
Coupling. J. Am. Chem. Soc..

[39] Gieshoff T., Schollmeyer D., Waldvogel S. R. (2016). Access to Pyrazolidin-3,5-diones
through Anodic N-N Bond Formation. Angew. Chem.
Int. Ed..

[40] Qian P., Zhou Z., Wang L., Wang Z., Wang Z., Zhang Z., Sheng L. (2020). Electrosynthesis of 2-(1,3,4-Oxadiazol-2-yl)­aniline
Derivatives with Isatins as Amino-Attached C1 Sources. J. Org. Chem..

[41] Yang N., Yuan G. (2018). A Multicomponent Electrosynthesis of 1,5-Disubstituted and 1-Aryl
1,2,4-Triazoles. J. Org. Chem..

[42] Klein M., Waldvogel S. R. (2022). Counter
Electrode Reactions—Important Stumbling
Blocks on the Way to a Working Electro-organic Synthesis. Angew. Chem. Int. Ed..

[43] Beil S. B., Pollok D., Waldvogel S. R. (2021). Reproducibility in Electroorganic
Synthesis-Myths and Misunderstandings. Angew.
Chem. Int. Ed..

[44] Hielscher M. M., Gleede B., Waldvogel S. R. (2021). Get into flow: Design of experiments
as a key technique in the optimization of anodic dehydrogenative C,C
cross-coupling reaction of phenols in flow electrolyzers. Electrochim. Acta.

[45] Weissman S. A., Anderson N. G. (2015). Design of Experiments (DoE) and Process Optimization.
A Review of Recent Publications. Org. Process
Res. Dev..

[46] Dörr M., Hielscher M. M., Proppe J., Waldvogel S. R. (2021). Electrosynthetic
Screening and Modern Optimization Strategies for Electrosynthesis
of Highly Value-added Products. ChemElectroChem.

[47] Francke R. (2022). Concepts for
sustainable organic electrosynthesis. Curr.
Opin. Electrochem..

[48] Titenkova K., Chaplygin D. A., Fershtat L. L. (2024). Electrochemical Generation of Nitrogen-centered
Radicals and its Application for the Green Synthesis of Heterocycles. ChemElectroChem.

