# Supplementary material for: Electrochemical Synthesis of Disubstituted Alkynes from Dihydrazones
Source: Org Lett. 2025 Jun 23;27(26):7053–7. doi: 10.1021/acs.orglett.5c01968 (PMC12235706; doi:10.1021/acs.orglett.5c01968)
Supplement: Supplementary file 1 [file ol5c01968_si_001.pdf]

Supporting Information

Electrochemical Synthesis of Disubstituted Alkynes from Dihydrazones

Subhabrata Dutta,[a] Jacob Kayser,[b] and Siegfried R. Waldvogel*[a,c]

[a] *Max-Planck-Institute for Chemical Energy Conversion (MPI CEC), Department of Electrosynthesis, Stiftstraße 34–36, 45470 Mülheim an der Ruhr (Germany)*

[b] *Department of Chemistry, Johannes Gutenberg University (JGU), Duesbergweg 10-14, 55128 Mainz (Germany)*

[c] *Karlsruhe Institute of Technology (KIT), Institute of Biological and Chemical Systems – Functional Molecular Systems (IBCS FMS), Kaiserstraße 12, 76131 Karlsruhe (Germany)*

Email: siegfried.waldvogel@cec.mpg.de

Table of Contents

1. GENERAL INFORMATION S3

1.1 General Information S3

1.2 Instruments and Analytical Methods S3

1.3 Electrochemical Setup S4

2. SCREENING AND OPTIMIZATION S7

2.1 Screening in a batch-type cell S7

2.2 Optimization using Design of Experiments (DoE) S10

3. CONTROL EXPERIMENTS S13

4. EXPERIMENTAL AND CHARACTERIZATION DATA S14

4.1 Starting material synthesis S14

4.2 General procedure for screening and optimization S19

4.3 General procedure for scale-up S19

4.4 General procedure for substrate scope S20

5. SCALABILITY TEST S25

6. REUSABILITY TEST S26

7. CYCLIC VOLTAMMETRY STUDIES S27

8. SPECTRA S28

9. REFERENCES S47

1. General Information

1.1 General Information

Starting materials and reagents were purchased from commercial suppliers (Sigma Aldrich, TCI, Alfa Aesar, Acros, BLD Pharma, and Fluka) and were used without further purification. Solvents were used as p.a. grade whereas high purity water was obtained by circulating deionized water through a Milli-Q® water purification system. Reactions were monitored by GC-FID. Analytical thin layer chromatography (TLC) was performed on silica gel 60 F254 aluminum plates (Merck).

1.2 Instruments and Analytical Methods

Chromatography

Thin layer chromatography (TLC) for reaction monitoring was performed using DC Kieselgel 60 F_{254} on aluminium plates (*Merck KGaA,* Darmstadt, Germany). A UV lamp (λ = 254 nm, UV-4 S/L, *Herolab GmbH Laborgeräte*, Wiesloch, Germany). TLC plates were visualized by exposure to short wave ultraviolet light (254 nm and 356 nm) and/or were dipped into a solution of $KMnO_4$ (1.5 g), K_2CO_3 (10.00 g) and KOH (1.5 mL of 10% solution) in H_2O (200 mL). Preparative column chromatography was performed manually on 100-200 mesh silica gel.

Gas Chromatography coupled with Mass Spectrometry (GC/MS)

Analysis of crude reaction mixtures and purified products were performed using a GCMS QP2010SE (*Shimadzu*, Kyoto, Japan) equipped with an electron ionization (EI) source and a quadrupole mass analyzer. A quartz capillary column HI-5MS (*Avantor VWR*, Radnor, USA) with the following specification was used: length of 30 m, inner diameter of 0.25 mm and a stationary phase (5%-phenyl-dimethylsiloxane) of 0.25 µm thickness. Helium was used as carrier gas with a constant velocity of 30 cm/s. The GC temperature ramp started at 50 °C (holding for 1 min) and heated to 300 °C (holding for 4.71 min) with a temperature ramp of 17.5 °C/min (total program time: 20.0 min). Measurements were performed at an injector temperature of 270 °C and a temperature of the EI source of 250 °C.

Nuclear Magnetic Resonance (NMR) Spectroscopy

^{1}H NMR and ^{13}C{^{1}H} NMR spectra were recorded at 25°C on a Bruker AVANCE III HD 400 MHz NMR spectrometer with a Bruker Prodigy probe (*Bruker BioSpin GmbH*, Rheinstetten, Germany) using $CDCl_3$ or DMSO as deuterated solvent. All chemical shifts are reported in δ-scale as parts per million [ppm] (multiplicity, coupling constant *J*, number of protons), relative to the solvent residual peaks as the internal standard. Coupling constants *J* are given in Hertz [Hz]. The following abbreviations were used to describe the signals: s (singlet), d (doublet), t (triplet), q (quartet), pent (pentet), sext (sextet), hept (heptet), m (multiplet), br (broad signal). The spectra obtained were evaluated with MestReNova 14 (*Mestrelab Research S.L.*, Spain).

Cyclic Voltammetry (CV) Measurements

Cyclic voltammetry was performed using a Metrohm 663 VA Stand equipped with an Autolab type III potentiostat (*Metrohm AG*, Herisau, Switzerland). WE: glassy carbon electrode (d = 2 mm); CE: glassy carbon rod; RE: Ag/$AgNO_3$; Scan rate v = 100 mV/s. Electrolyte: NBu_4PF_6 (0.1 mol/L) in acetonitrile; c(substrate) = 10 mmol/L; V = 5 mL. All solutions have been deoxygenated by bubbling with argon for 10 min prior to measurement. Electrodes have been thoroughly rinsed before and after each measurement with acetone and acetonitrile. All data is displayed against the half-wave potential of ferrocene/ferrocenium redox couple (FcH/FcH^+; –0.10 V vs. Ag/$AgNO_3$) as internal reference. Oxidation potentials are marked and displayed as the half-wave potential of the respective peak.[1–3]

1.3 Electrochemical Setup

Galvanostat (DC power source)

Electrochemical reactions were carried out using a multichannel galvanostat HMP4040 programmable power supply (4 channels per device; max. electric current per channel: 10 A; max. power per channel: 160 W; total output power per device: 384 W; upper terminal voltage limit per channel: 32 V; *Rohde & Schwarz GmbH & Co.* KG, Munich, Germany). The experiments were performed under galvanostatic conditions using a simple two-electrode reaction setup.

Screening and small-scale batch-type reactions

Screening reactions were carried out in undivided Teflon™ cells with a volume of 10 mL equipped with two electrodes and a round, cross-shaped stirring bar. The described system is commercially available as IKA

Screening System Package (*IKA™ Werke GmbH & Co. KG*, Staufen, Germany). Detailed description of these cells are reported in the literature.[4]

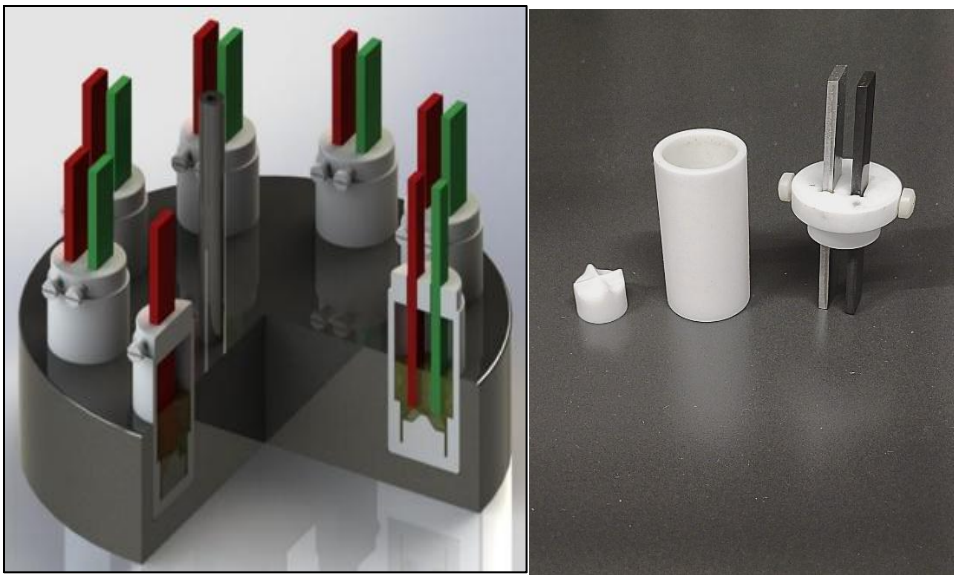

Figure S1: a) Undivided screening setup as available from IKA equipped with graphite as anode and stainless steel as cathode; b) screening cell (undivided) equipped with stirring bar, electrode holder, and two electrodes (graphite and stainless steel).[5]

The screening was carried out using various electrode materials (electrode size: 7 cm × 1 cm × 0.3 cm) (Table S1). Graphite electrodes were sanded using sandpaper of grit size 600, followed by grit size 1000, rinsed with water, and cleaned with acetonitrile/acetone prior to use.

Table S1: Electrode materials, specification, and supplier.

Entry	Electrode Material	Specification	Supplier
1	Graphite	Highly isostatic, V2100	*SGL Carbon*, Bonn, Germany
2	Stainless steel	1.4571	various suppliers (metal traders)
3	Nickel	-	*IKA Werke GmbH & Co*. KG, Germany
4	Glassy Carbon	Sigradur G	*HTW*, Thierhaupten, Germany
5	Platinum	99.9% Pt	*ÖGUSSA*, Vienna, Austria.
6	Graphite foil (Sigraflex™)	F02012Z	*SGL Carbon*, Meitingen, Germany

7	BDD	DIACHEM™ 15 µm diamond layer on silicon	*CONDIAS* GmbH, Germany

Scale-up

The scale up experiment was performed on two large scales, 2,5 mmol and 15.0 mmol. The former requires a double-walled undivided glass cell with no thermostat attached, with a volume of 25 mL equipped with a PTFE stopper and sleeve, electrodes, electrode holders and a cross-shaped stirring bar (Figure S2A). The latter is a single-walled undivided cell equipped with PTFE stopper, electrodes, electrode holders, and circular stirring bar (Figure S2B). The cell is commercially available *via HWS Labortechnik* (Mainz, Germany) as Sigma-Aldrich within the SynLectro™ series. The electrolysis was conducted with multichannel galvanostat HMP4040. The electrode dimensions were 6 cm × 2 cm × 0.02 cm and was submerged into the solution (3.5 cm depth). This resulted in an active electrode area of 7 cm^2. The stoppers and electrode holders are available from Sigma-Aldrich within the SynLectro™ series.

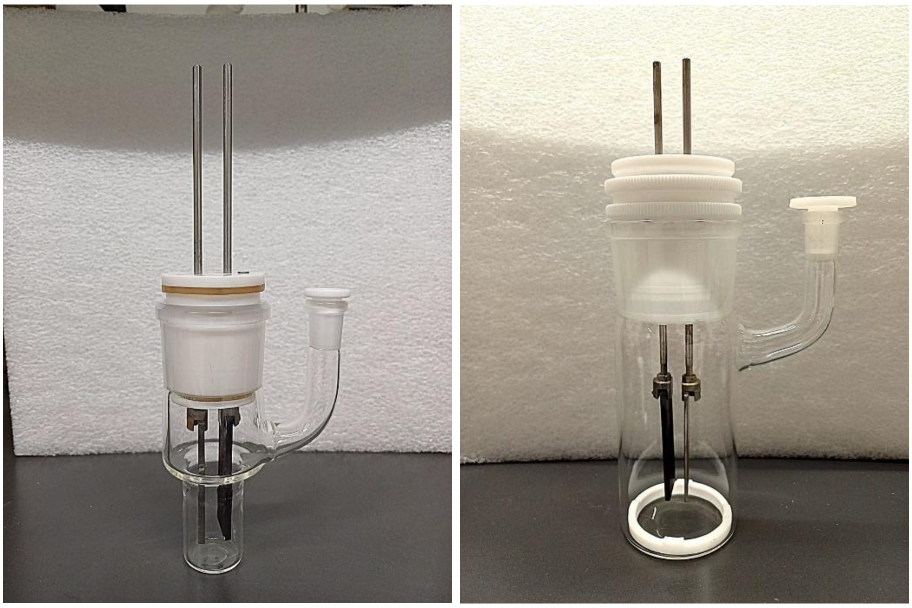

Figure S2: A) 25 mL undivided cell equipped with graphite anode, stainless steel cathode, and electrode holders used for 2.25 mmol scale (*left*); B) 100 mL undivided cell equipped with graphite anode, stainless steel cathode, and electrode holders used for 15.0 mmol scale (*right*). Same sized electrodes were used in both cases.

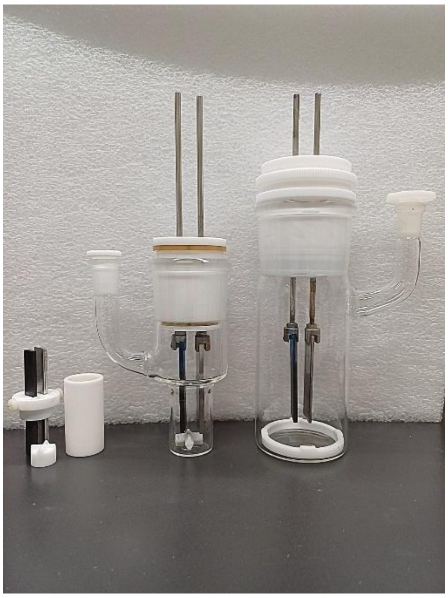

Figure S3: Comparative image for all three electrolysis cells used in the project.

2. Screening and Optimization

2.1 Screening in a batch-type cell

Optimization of the reaction conditions was carried out by using a linear screening approach as well as Design of experiments. Compound **1a** was chosen for the optimization purpose.

General procedure (GP-1): The reactions are carried out using the undivided Teflon™ cells with a PTFE lid including the anode and a cathode as described in the section before.

An undivided Teflon™ cell is filled with methanol (7 mL), followed by the addition of supporting electrolytes and additive (*quantities are given in section 3*). The reaction mixture was vigorously stirred to ensure maximum solubility. Then **1a** (0.5 mmol, 0.07 M) is added to the reaction mixture and stirred again for solubility aspect. The electrodes were fixed in the lid with screws, ensuring the correct height of electrodes dipped for accurate current density. The amperage and amount of applied charge are set to the desired values, and the electrolysis was carried out at room temperature under constant stirring. After the required period of time, 1,3,5-trimethoxybenzene (60 mg) is as added to the reaction mixture as an internal standard. The solution is stirred for 5 min at room temperature to make sure the internal standard is well mixed homogenously. A GC sample is prepared using the reaction mixture and yield is calculated based on the respective area on the GC spectra. Each experiment was done twice and the average yield of two experiments are reported.

Initial experiment:

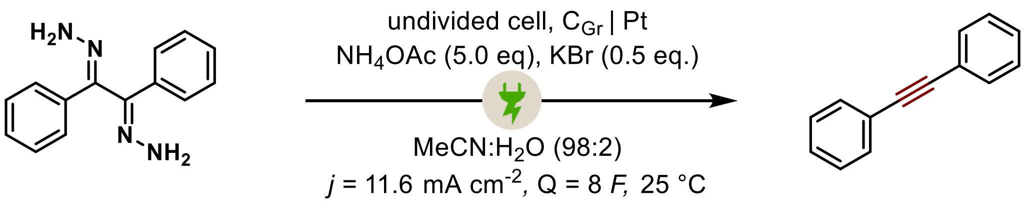

1a, 0.5 mmol (0.07 M) **2a**, 43%

Initial experiments were carried out in an undivided Teflon™ cell with a graphite anode and platinum cathode with NH_4OAc as supporting electrolyte.

Next, different combinations of solvent, additive, and electrolyte systems were tested.

Table S2: Combination of solvent, electrolyte, and additive systems

Entry	Solvent	Electrolyte (eq.)	Additive (0.5 eq)	Yield (GC-FID)
**1**	MeCN:H_2O (98:2)	NH_4OAc (5.0)	KBr	43%
**2**	MeCN:H_2O (98:2)	NH_4OAc (5.0)	NaBr	37%
**3**	MeCN:H_2O (98:2)	NH_4OAc (5.0)	NH_4Br	32%
**4**	MeCN:AcOH (90:10)	KBr (0.5)	–	8%
**5**	MeCN:AcOH (90:10)	HBr in AcOH (0.5)	–	8%
**6***	MeCN:AcOH (90:10)	KOAc (0.5)	–	32%

Conditions *(GP-1): substrate (0.5 mmol, 0.07 M), j = 11.6 mA cm^{-2}, 8 F, 3 mm interelectrode gap, 450 rpm. rt*

*Given that KOAc alone produced results comparable to those obtained with the combination of NH_4OAc and additives, we proceeded with KOAc while omitting the additional components.

Next, we tested the various solvent combinations with KOAc as electrolyte.

Table S3: Combination of solvents and amount of KOAc

Entry	Solvent (ratio)	KOAc (eq.)	Yield % (GC-FID)
**1**	MeCN:AcOH (99:1)	1	49
**2**	MeCN:AcOH (95:5)	1	51
**3**	MeCN:AcOH (90:10)	1	22
**4**	MeCN:AcOH (90:10)	2	36

**5**	MeCN:AcOH (90:10)	3	37
**6**	MeCN:AcOH (90:10)	4	38
**7**	MeCN:AcOH:MeOH (90:5:5)	2	50
**8**	MeCN:AcOH:EtOH (90:5:5)	2	5
**9**	AcOH:EtOH (95:5)	2	50
**10**	EtOH	2	53
**11**	MeOH	2	73

***Conditions** (GP-1): substrate (0.5 mmol, 0.07 M), j = 11.6 mA cm^{-2}, 8 F, 3 mm interelectrode gap, 450 rpm. rt*

Table S4: Anode testing

Entry	Anode	KOAc (eq.)	Yield (GC-FID)
**1**	Stainless steel	2	11%
**2**	Nickel	2	24%
**3**	BDD	2	0%
**4**	Sigraflex	2	19%
**5**	Platinum	2	26%
**6**	Glassy carbon	2	3%

***Conditions** (GP-1): substrate (0.5 mmol, 0.07 M), MeOH as solvent, j = 11.6 mA cm^{-2}, 8 F, 3 mm interelectrode gap, 450 rpm. rt*

Table S5: Screening of stirring rate.

Entry	Rotations per min (rpm)	Yield (GC-FID)
**1**	100	67%
**2**	450	73%
**3**	750	76%
**4**	1000	74%

***Conditions** (GP-1): substrate (0.5 mmol, 0.07 M), MeOH as solvent, j = 11.6 mA cm^{-2}, 8 F, 3 mm interelectrode gap, rt*

Conclusion: MeOH/KOAc combination works the best. The electrochemical parameters are now optimized using DoE (design of experiments).

2.2 Optimization using Design of Experiments (DoE)

The effect of selected continuous reaction parameters on reaction yield was evaluated using the Design of Experiments (DoE) approach.[6,7] The experimental design was planned and evaluated with the aid of S14 Minitab 19.2020.1 (Minitab LLC). The DoE experiments were carried out in 25 mL double-layered tempered glass cells. This experiment was used to screen the amount of substrate, concentration of KOAc, temperature, amount of applied charge, and current density.

Table S6: Range of parameters ($2^{(5-2)}$ design)

Parameter	Lower limit	Upper limit
Amount of substrate (mmol)	0.75	2.25
Concentration of KOAc (M)	0.04	1.0
Temperature (°C)	25	60
Amount of applied charge (F)	4	12
Current density (mA cm^{-2})	5.5	16.5

Conditions (GP2): MeOH as solvent, 3 mm interelectrode gap

The experiments were conducted according to the general procedure 1 (GP-1). After each experiment the electrodes were washed as explained in the general procedure. In case we observed bigger deviations in yield (more than 8%), we repeated the experiment two more times and crossed out the point which was furthest away from the average value.

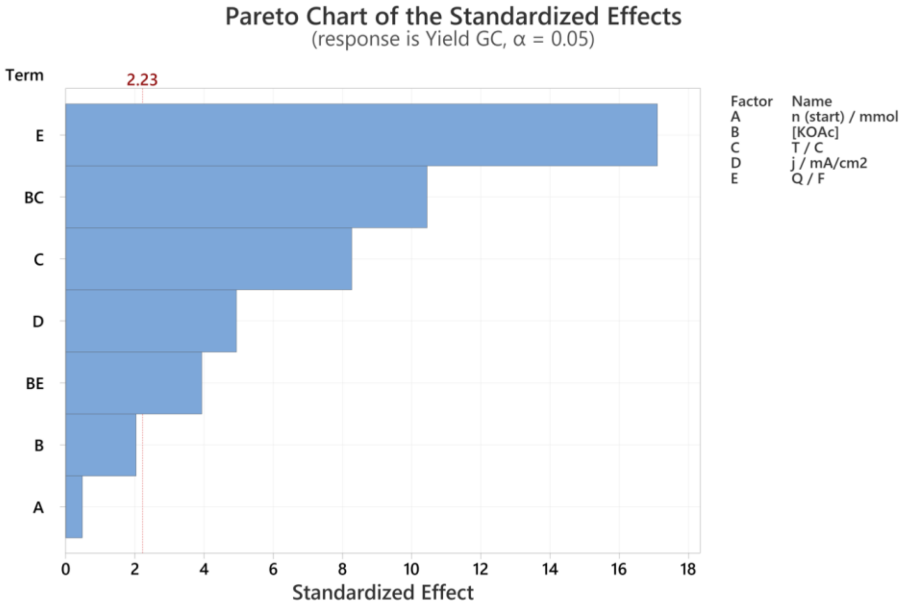

Figure S4. Pareto chart for analyzing the individual and synergistic effects of different parameters.

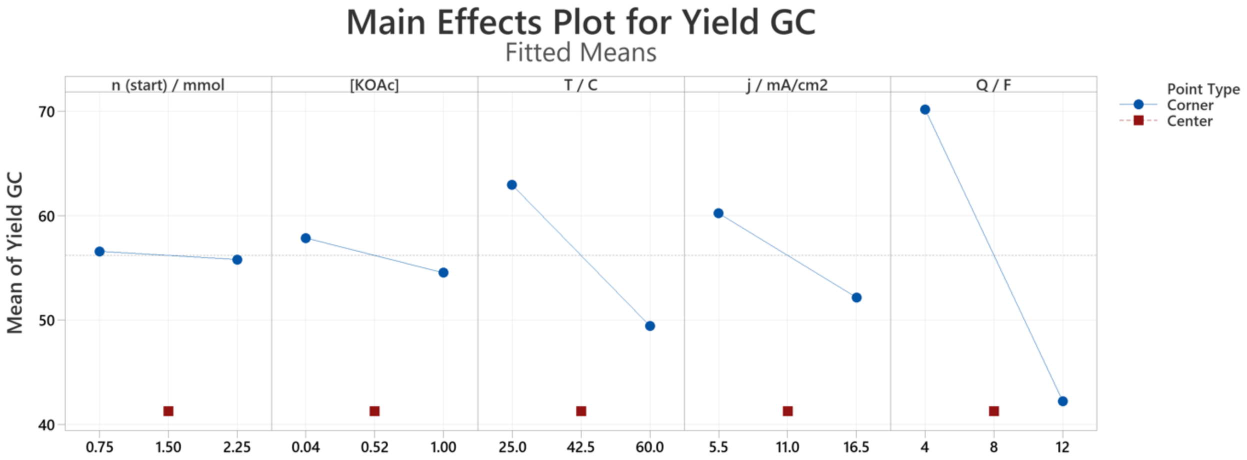

Figure S5. Trends for individual parameters.

Conclusion: The main plot shows that the amount of substrate is not having a significant role in the yield of the reaction. Moreover, for the ease of reaction setup, we chose to operate with 25 °C. Next, we further narrowed down the DoE using a $2^{(3-1)}$ design, with constant T (°C) and n (mmols).

Table S7: Range of parameters ($2^{(3-1)}$ design).

Parameter	Lower limit	Upper limit
Concentration of KOAc (M)	0.07	0.5
Amount of applied charge (F)	4	5
Current density (mA cm^{-2})	5	7

Conditions (GP2): substrate (2.25 mmol, 0.15 M), MeOH as solvent, 3 mm interelectrode gap, 25 °C,

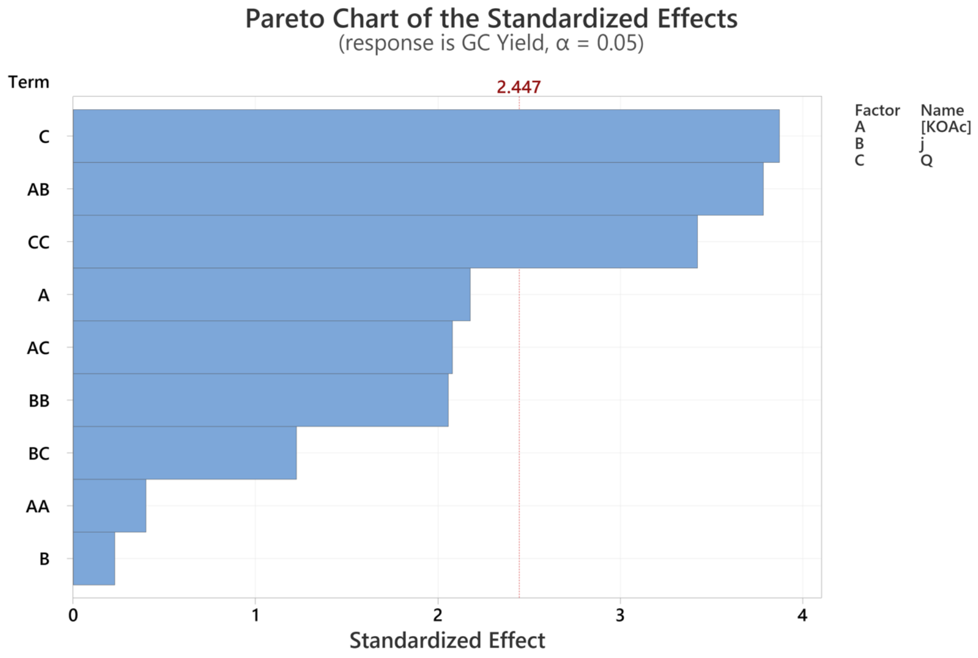

Figure S6. Pareto chart for analyzing the individual and synergistic effects of different parameters.

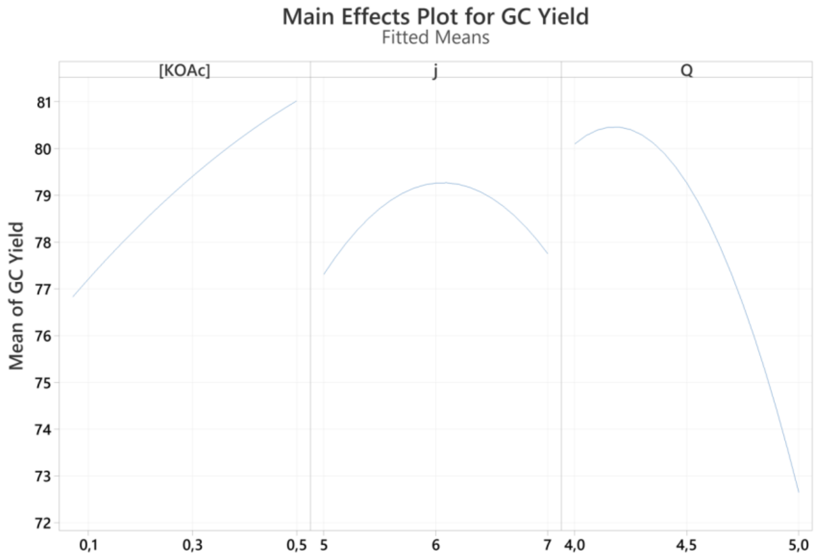

Figure S7. Trends for individual parameters

Trends in parameters: To address reproducibility concerns, we evaluated the variation in key reaction parameters (Table S8). Using the optimized reaction conditions as a reference point, we systematically tested both lower and higher values to span a representative range.

Table S8: Final optimized reaction conditions.

Parameters and Deviations		
Temperature	10 °C	72%
	50 °C	58%
Stirring speed	500 rpm	72%
	1000 rpm	74%
[KOAc]	0.07 M	52%
	0.7 M	63%

Table S9: Final optimized reaction conditions for substrate **1a**.

c(SM)	Supporting electrolyte (SE)	*c*(SE)	*j* [mA cm^{-2}]	Cathode material	Anode material	*T*	*Q* [*F*]	Yield 1a (GC)
0.15	KOAc	0.5	6.0	C_{Gr}	Stainless steel	25 °C	4	81%

3. Control Experiments

Table S10: Control experiments with optimized conditions.

Entry	Deviation from the standard conditions[a]	Yield (^{1}H NMR)
1	No electricity; stirred for 6 h at room temperature	0%

4. Experimental and Characterization Data

4.1 Starting material synthesis

Synthesis of diketones:

1,2-diketones were prepared using a modified literature procedure.[8] A mixture of the starting ketone (10 mmol) and sodium nitrite (2.07 g, 30 mmol) was suspended in 20 mL of tetrahydrofuran (THF) and cooled to 0 °C. Concentrated hydrochloric acid (13 mL, 37% w/w) was then added dropwise to the suspension with stirring, ensuring the temperature remained below 10 °C throughout the addition. Upon completion of the acid addition, the cooling bath was removed, and the mixture was left overnight at ambient temperature. The reaction's progress was tracked using gas chromatography (GC). Once the starting ketone was fully consumed, the reaction mixture—containing the crude 1,2-diketone—was transferred into a separatory funnel containing 50 g of crushed ice and 100 mL of diethyl ether. The organic phase was separated, and the aqueous phase was extracted three additional times with diethyl ether (3 × 100 mL). All ether extracts were combined, washed successively with a saturated aqueous solution of sodium bicarbonate (100 mL) and brine (100 mL), then dried over magnesium sulfate. After filtration and evaporation under reduced pressure, the crude product was purified by filtration through a silica gel pad using *n*-pentane: diethyl ether as eluent.

3-Methyl-1-phenylbutane-1,2-dione

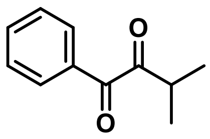

The characteristic data matched with the experimental precedence data.[9]

The compound was obtained as yellow oil.

R$_f$: 0.7 (92:8 pentane:diethyl ether)

Yield: 43%

^{1}H NMR (CDCl3, 400 MHz) δ 7.93 (d, J = 7.6 Hz, 2 H), 7.44 – 7.62 (m, 3 H), 3.37 (q, J = 6.8 Hz, 1 H), 1.20 (q, J = 6.8 Hz, 6 H);

^{13}C NMR (CDCl3, 100 MHz) δ 207.5, 194.3, 134.8, 133.8, 130.5, 129.1, 128.8, 128.1, 37.2, 17.3.

Synthesis of dihydrazones (*General procedure 2– GP2*):

The procedure is adapted from a modified literature precedence.[10] The 1,2-dicarbonyl (1.0 equiv.) and ethanol (0.8 M) are added to a suitable pressure tube (or Schlenck tube) equipped with a PTFE-coated stirring bar. Hydrazine hydrate (10 eq.) is added to the mixture and the pressure tube is sealed with a Teflon screw cap. The mixture is heated at 100 °C for 2 days until the substrate and monohydrazone are no longer detected via gas chromatography. After cooling to room temperature, the crystalline dihydrazone is filtered and dried under suction. Water (100 mL) is added to the filtered solution to precipitate the remaining product and monohydrazone, which are then filtered.

1,2-Diphenylethane-1,2-dione dihydrazone (1a)

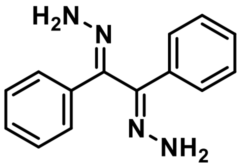

The title compound was synthesized using diphenyl-1,2-dione and hydrazine hydrate using GP-2. The compound was obtained as colorless solid. *The spectral data matches with the precedented literature.*[10]

Yield: 80%

^{1}H NMR (400 MHz, CD_2Cl_2) δ 7.61 – 7.51 (m, 4H), 7.35 – 7.25 (m, 6H), 5.81 (s, 4H).

^{13}C NMR (101 MHz, CD_2Cl_2) δ 142.2, 135.4, 129.1, 129.0, 125.9.

1,2-Di(4-methylphenyl)-1,2-ethanedione 1,2-dihydrazone (1b)

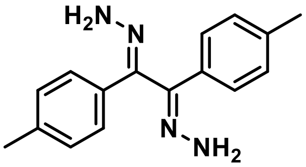

The title compound was synthesized using di-*p*-tolylethane-1,2-dione and hydrazine hydrate using GP-2. The compound was obtained as colorless solid.

Yield: 78%

^{1}H NMR (400 MHz, CD_2Cl_2) δ 7.46 – 7.38 (m, 4H), 7.12 (d, J = 8.0 Hz, 4H), 5.70 (s, 4H), 2.31 (s, 6H).

^{13}C NMR (101 MHz, CD_2Cl_2) δ 142.7, 139.2, 132.6, 129.8, 125.9, 21.4.

GCMS (EI) m/z: calc'd for C16H18N4 266.1526; found 266.1529.

1,2-Di(4-*tert*-butyl phenyl)-1,2-ethanedione 1,2-dihydrazone (1c)

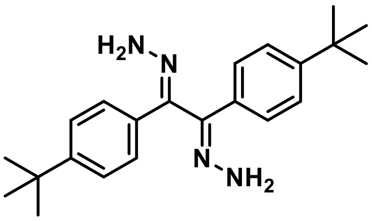

The title compound was synthesized using 1,2-di(4-(*tert*-butyl)phenyl)ethane-1,2-dione and hydrazine hydrate using GP-2. The compound was obtained as colorless solid.

Yield: 56%

^{1}H NMR (400 MHz, $CDCl_3$) δ 7.55 – 7.47 (m, 4H), 7.40 – 7.30 (m, 4H), 1.29 (s, 18H).

^{13}C NMR (101 MHz, $CDCl_3$) δ 152.1, 143.0, 131.9, 125.9, 125.4, 77.4, 34.8, 31.4.

GCMS (EI) m/z: calc'd for C22H30N4 350.2465; found 350.2468.

1,2-Di(4-fluorophenyl)-1,2-ethanedione 1,2-dihydrazone (1d)

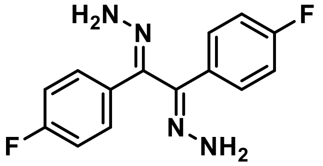

The title compound was synthesized using 1,2-di(4-fluorophenyl)ethane-1,2-dione and hydrazine hydrate using GP-2. The compound was obtained as off-white solid.

Yield: 69%

^{1}H NMR (400 MHz, CD_2Cl_2) δ 7.58 – 7.45 (m, 4H), 7.06 – 6.97 (m, 4H), 5.79 (s, 4H).

^{13}C NMR (101 MHz, CD_2Cl_2) δ 164.7, 162.2, 152.5, 131.5 (d, *J* = 3.2 Hz), 127.7 (d, *J* = 8.2 Hz), 116.0 (d, *J* = 21.8 Hz).

^{19}F NMR (376 MHz, CD_2Cl_2) δ -112.5.

GCMS (EI) m/z: calc'd for C14H12F2N4 274.1025; found 274.1028.

1,2-Di(4-chlorophenyl)-1,2-ethanedione 1,2-dihydrazone (1e)

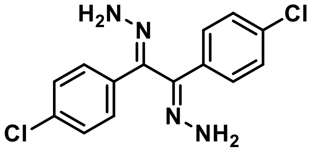

The title compound was synthesized using 1,2-di(4-chlorophenyl)ethane-1,2-dione and hydrazine hydrate using GP-2. The compound was obtained as colorless solid.

Yield: 77%

^{1}H NMR (400 MHz, CD_2Cl_2) δ 7.6 – 7.4 (m, 4H), 7.3 – 7.2 (m, 4H), 5.9 (s, 4H).

^{13}C NMR (101 MHz, CD_2Cl_2) δ 140.1, 134.6, 133.8, 129.3, 127.2.

GCMS (EI) m/z: calc'd for C14H12Cl2N4 (isotope ^{35}Cl) 306.0434; found 306.0434.

1,2-Di(4-methoxyphenyl)-1,2-ethanedione 1,2-dihydrazone (1f)

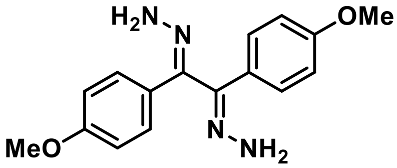

The title compound was synthesized using 1,2-di(4-methoxyphenyl)ethane-1,2-dione and hydrazine hydrate using GP-2. The compound was obtained as colorless solid.

Yield: 55%

^{1}H NMR (400 MHz, CD_2Cl_2) δ 7.52 – 7.42 (m, 4H), 6.88 – 6.80 (m, 4H), 5.64 (s, 4H), 3.77 (s, 6H).

^{13}C NMR (101 MHz, CD_2Cl_2) δ 160.6, 142.7, 127.9, 127.3, 114.5, 55.7.

GCMS (EI) m/z: calc'd for C16H18N4O2 298.1424; found 298.1426.

1,2-Di(3-methylphenyl)-1,2-ethanedione 1,2-dihydrazone (1g)

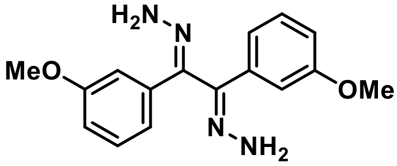

The title compound was synthesized using 1,2-di(3-methoxyphenyl)ethane-1,2-dione and hydrazine hydrate using GP-2. The compound was obtained as colorless solid.

Yield: 59%

^{1}H NMR (400 MHz, $CDCl_3$) δ 7.24 – 7.17 (m, 4H), 7.04 (ddd, J = 7.7, 1.6, 1.0 Hz, 2H), 6.84 (ddd, J = 8.2, 2.7, 1.0 Hz, 2H), 5.62 (s, 4H), 3.80 (s, 6H).

^{13}C NMR (101 MHz, $CDCl_3$) δ 160.1, 142.1, 136.2, 129.9, 118.5, 115.2, 110.1, 55.4.

GCMS (EI) m/z: calc'd for C16H18N4O2 298.1424; found 298.1427.

1,2-Di(2-methoxyphenyl)-1,2-ethanedione 1,2-dihydrazone (1h)

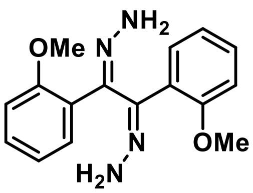

The title compound was synthesized using 1,2-di(2-methoxyphenyl)ethane-1,2-dione and hydrazine hydrate using GP-2.

Yield: 55%

^{1}H NMR (400 MHz, $CDCl_3$) δ 7.40 (ddd, *J* = 8.1, 7.3, 1.9 Hz, 2H), 7.17 (dd, *J* = 7.4, 1.8 Hz, 2H), 7.14 – 7.01 (m, 4H), 5.28 (s, 4H), 3.85 (s, 6H).

^{13}C NMR (101 MHz, $CDCl_3$) δ 156.9, 148.6, 130.9, 130.4, 122.2, 121.4, 112.0, 56.2.

GCMS (EI) m/z: calc'd for C16H18N4O2 298.1424; found 298.1425.

1-Phenyl-1,2-propanedione 1,2-dihydrazone (1i)

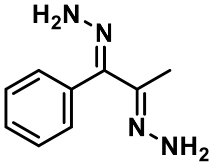

The title compound was synthesized using 1-phenylpropane-1,2-dione and hydrazine hydrate using GP-2. The compound was obtained as crystalline colorless solid.

Yield: 71%

^{1}H NMR (400 MHz, CD_2Cl_2) δ 7.48 – 7.42 (m, 2H), 7.39 – 7.34 (m, 1H), 7.17 – 7.09 (m, 2H), 5.35 – 5.29 (m, 4H), 2.05 (s, 3H).

^{13}C NMR (101 MHz, CD_2Cl_2) δ 150.2, 148.8, 133.3, 129.2, 129.1, 128.6, 9.1.

GCMS (EI) m/z: calc'd for C9H12N4 176.1056; found 176.1057.

3-Methyl-1-phenylbutane-1,2-dione 1,2-dihydrazone (1j)

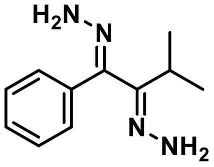

The title compound was synthesized using 3-methyl-1-phenylbutane-1,2-dione and hydrazine hydrate using GP-2. The compound was obtained as off-white solid.

Yield: 66%

^{1}H NMR (400 MHz, CD_2Cl_2) δ 7.51 – 7.41 (m, 2H), 7.38 – 7.28 (m, 3H), 5.75 (s, 2H), 5.39 (s, 2H), 2.51 (hept, *J* = 6.9 Hz, 1H), 1.14 (d, *J* = 6.8 Hz, 3H), 0.99 (d, *J* = 7.0 Hz, 3H).

^{13}C NMR (101 MHz, CD_2Cl_2) δ 150.5, 144.4, 135.7, 129.0, 128.8, 126.2, 54.3, 54.0, 53.8, 33.2, 20.3, 19.2.

GCMS (EI) m/z: calc'd for C11H16N4 204.1369; found 204.1370.

2,3-Heptanedione, 2,3-dihydrazone (1k)

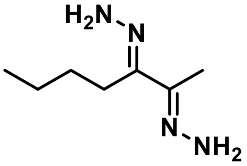

The title compound was synthesized using heptane-2,3-dione and hydrazine hydrate using GP-2. The compound was obtained as needle-like colorless solid.

Yield: 82%

¹H NMR (400 MHz, CD_2Cl_2) δ 5.48 – 5.22 (m, 4H), 2.55 – 2.44 (m, 2H), 1.88 (s, 3H), 1.46 – 1.28 (m, 4H), 0.98 – 0.85 (m, 3H).

¹³C NMR (101 MHz, CD_2Cl_2) δ 151.9, 147.8, 27.8, 23.6, 22.7, 14.1, 8.5.

GCMS (EI) m/z: calc'd for C7H16N4 156.1369; found 156.1370.

4.2 General procedure for screening and optimization

The reactions are carried out using the undivided Teflon™ cells with a PTFE lid including the anode and a cathode as described in the section before.

An undivided Teflon™ cell is filled with methanol (7 mL), followed by the addition of supporting electrolytes and additive (*quantities are given in section 2*). The reaction mixture was vigorously stirred to ensure maximum solubility. Then **1a** (0.5 mmol, 0.07 M) is added to the reaction mixture and stirred again for solubility aspect. The electrodes were fixed in the lid with screws, ensuring the correct height of electrodes dipped for accurate current density. The amperage and amount of applied charge are set to the desired values, and the electrolysis was carried out at room temperature under constant stirring. After the required period, 1,3,5-trimethoxybenzene (60 mg) is as added to the reaction mixture as an internal standard. The solution is stirred for 5 min at room temperature to make sure the internal standard is well mixed homogenously. A GC sample is prepared using the reaction mixture and yield is calculated based on the respective area on the GC spectra.

4.3 General procedure for scale-up

The reaction is carried out using the single wall undivided glass cell with a graphite anode and stainless steel as a cathode as described in the section above. A circular stir bar was used with lower stirring rate to avoid formation of a strong vortex. This ensured the continuous contact of electrodes with the solution.

The glass cell is filled with methanol (100 mL), followed by the addition of KOAc (4.9 g, 0.5 M). The reaction mixture was vigorously stirred to ensure maximum solubility. Then **1a** (3.57 g, 15 mmol, 0.07 M) is added to the reaction mixture and stirred again for solubility aspect. The electrodes were fixed in the PTFE stopper, ensuring the correct height of electrodes dipped for accurate current density (6 mA cm^{-2}). The amperage (42 mA) and amount of applied charge (5789 C) are set to the desired values, and the electrolysis was carried out at room temperature under constant stirring. After the required period of time, 1,3,5-trimethoxybenzene (2.52 g) is as added to the reaction mixture as an internal standard. The solution is stirred for 5 min at room temperature to make sure the internal standard is well mixed homogenously. A GC sample is prepared using the reaction mixture and yield is calculated based on the respective area on the GC spectra.

Spectroscopic data of the compound matched the one obtained from the small-scale reaction described in next section.

4.4 General procedure for substrate scope

General procedure (GP-3): The reactions are carried out using the undivided single-wall tempered glass cell as described in the section before. Graphite and stainless steel were chosen as the anode and the cathode respectively. The electrodes were cleaned before use. The graphite was rubbed with 1000 grit size sandpaper, whereas the stainless steel was treated with a 0.1 M HCl solution, followed by washing with water and drying.

The dihydrazone (2.25 mmol) and potassium acetate (0.5 M, 7.5 mmol, 736 mg, 3.3 eq.) are suspended in methanol (15 mL) in an undivided, tempered, glass beaker-type cell, equipped with a reflux condenser. The electrolysis is conducted using a graphite anode and a stainless-steel cathode, using 4 *F* (869 C), a current density of 6 mA cm^{-2} (I = 42 mA, I = j×A, A = electrode area and j = current density), a stirring speed of 750 rpm and a temperature of 25 °C. Ethyl acetate (50 mL) is added to the resulting solution and is washed three times with water (20 mL) and once with brine (20 mL). The organic phase is dried using magnesium sulphate, dried under vacuum, and further purified by column chromatography. The chromatography conditions are listed against the substrate scope.

Note 1: The substrate is usually not completely dissolved in the electrolyte solution, and stirring should be ensured before electrolysis is carried out.

Note 2: The immersion depth of the electrodes was adjusted based on the stirring conditions. The electrodes were always positioned as close to the stirrer as possible without causing disturbances. With electrode dimensions of 2 cm by 6 cm, an immersion depth of 3.5 cm was measured, resulting in an immersed electrode surface area of 7 cm^2. Depending on the cell, the electrode mounts were further stabilized by wrapping Teflon tape around the top of the electrodes.

Tolane (2a)

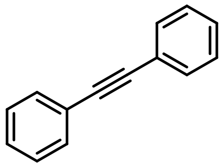

The title compound was synthesized with **1a** using GP-3 on a 2.25 mmol scale. The title compound was obtained as colorless solid. *The analytical data matches with the literature precedence.*[12]

R_f: 0.9 (100% pentane)

Yield: 320 mg, 80%

^{1}H NMR (400 MHz, $CDCl_3$) δ 7.56 – 7.54 (m, 4H), 7.39 – 7.32 (m, 6H).

^{13}C NMR (101 MHz, $CDCl_3$) δ 131.6, 128.3, 128.2, 123.3, 89.4.

4,4'-Dimethyl tolane (2b)

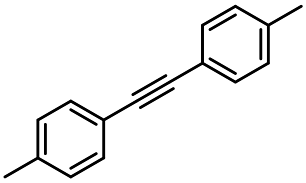

The title compound was synthesized with **1b** using GP-3 on a 2.25 mmol scale. *The analytical data matches with the literature precedence.*[11]

Rf: 0.9 (100% pentane)

Yield: 361 mg, 78%

^{1}H NMR (400 MHz, $CDCl_3$) δ 7.46 – 7.40 (m, 4H), 7.18 – 7.13 (m, 4H), 2.37 (s, 6H).

^{13}C NMR (101 MHz, $CDCl_3$) δ 138.3, 131.6, 129.2, 120.5, 89.0, 21.6.

4,4'-Di-*tert*-butyl tolane (2c)

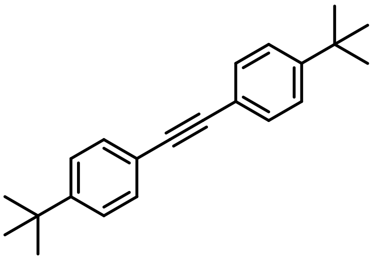

The title compound was synthesized with **1c** using GP-3 on 2.25 mmol scale. The title compound was obtained as colorless amorphous solid. *The analytical data matches with the literature precedence.*[11]

Rf: 0.9 (100% pentane)

Yield: 274 mg, 42%

^{1}H NMR (400 MHz, $CDCl_3$) δ 7.56 – 7.45 (m, 4H), 7.43 – 7.33 (m, 4H), 1.4 (s, 18H).

^{13}C NMR (101 MHz, $CDCl_3$) δ 151.4, 131.4, 125.4, 120.6, 89.0, 34.9, 31.3

4,4'-Difluorotolane (2d)

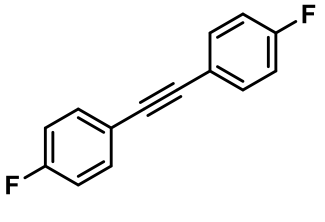

The title compound was synthesized with **1d** using GP-3 on 2.25 mmol scale. The title compound was obtained as colorless solid. *The analytical data matches with the literature precedence.*[13]

Rf: 0.9 (100% pentane)

Yield: 355 mg, 74%

^{1}H NMR (400 MHz, $CDCl_3$) δ 7.56 – 7.45 (m, 4H), 7.10 – 7.00 (m, 4H).

^{13}C NMR (101 MHz, $CDCl_3$) δ 163.9, 161.4, 133.6 (d, J = 8.4 Hz), 115.8 (d, J = 22.1 Hz), 88.1.

^{19}**F NMR** (376 MHz, $CDCl_3$) δ -110.8.

4,4'-Dichlorotolane (2e)

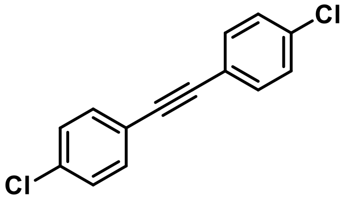

The title compound was synthesized with **1e** using GP-3 on 2.25 mmol scale. The title compound was obtained as colorless crystalline solid. *The analytical data matches with the literature precedence.*[14]

R$_f$: 0.9 (100% pentane)

Yield: 393 mg, 71%

^{1}**H NMR** (400 MHz, $CDCl_3$) δ 7.49 – 7.40 (m, 1H), 7.37 – 7.29 (m, 1H).

^{13}**C NMR** (101 MHz, $CDCl_3$) δ 134.7, 132.9, 128.9, 121.6, 89.3.

4,4'-Di(4-methoxy)tolane (2f)

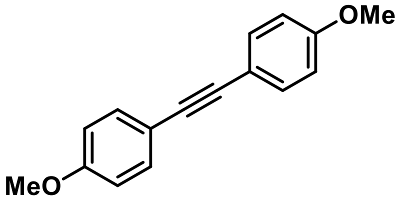

The title compound was synthesized with **1f** using GP-3 on 2.25 mmol scale. The title compound was obtained as colorless solid. *The analytical data matches with the literature precedence.*[11]

R$_f$: 0.8 (95:5 pentane:diethyl ether)

Yield: 283 mg, 53%

^{1}**H NMR** (400 MHz, $CDCl_3$) δ 7.50 – 7.42 (m, 4H), 6.92 – 6.84 (m, 4H), 3.82 (s, 6H).

^{13}**C NMR** (101 MHz, $CDCl_3$) δ 159.5, 133.0, 115.8, 114.1, 88.1, 55.4.

4,4'-Di(3-methoxy)tolane (2g)

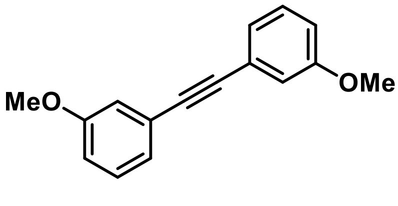

The title compound was synthesized with **1g** using GP-3 on 2.25 mmol scale. The title compound was obtained as colorless solid. *The analytical data matches with the literature precedence.*[15]

R$_f$: 0.8 (93:7 pentane:diethyl ether)

Yield: 293 mg, 55%

^{1}H NMR (400 MHz, $CDCl_3$) δ 7.30 – 7.23 (m, 2H), 7.15 (dt, *J* = 7.6, 1.2 Hz, 2H), 7.08 (dd, *J* = 2.7, 1.4 Hz, 2H), 6.91 (ddd, *J* = 8.3, 2.7, 1.1 Hz, 2H), 3.83 (s, 6H).

^{13}C NMR (101 MHz, $CDCl_3$) δ 159.5, 129.6, 124.3 (d, *J* = 4.0 Hz), 116.5, 115.1, 89.3, 55.4.

4,4'-Di(2-methoxy)tolane (2h)

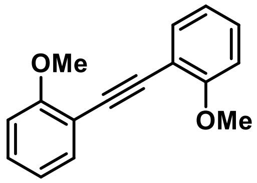

The title compound was synthesized with **1h** using GP-3 on 2.25 mmol scale. The title compound was obtained as colorless solid. *The analytical data matches with the literature precedence.*[11]

R_f: 0.7 (95:5 pentane:diethyl ether)

Yield: 259 mg, 48%

^{1}H NMR (400 MHz, $CDCl_3$) δ 7.53 (dd, *J* = 7.6, 1.7 Hz, 2H), 7.33 – 7.26 (m, 2H), 6.96 – 6.87 (m, 4H), 3.93 (s, 6H).

^{13}C NMR (101 MHz, $CDCl_3$) δ 160.0, 133.7, 129.7, 120.6, 113.0, 110.8, 90.0, 56.1.

Methylphenylacetylene (2i)

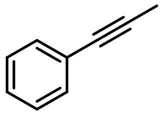

The title compound was synthesized with **1i** using GP-3 on 2.25 mmol scale. The title compound was obtained as colorless oil. *The analytical data matches with the literature precedence.*[16]

R_f: 0.9 (100% pentane)

Yield: 116 mg, 60%

^{1}H NMR (400 MHz, $CDCl_3$) δ 7.44 – 7.37 (m, 2H), 7.33 – 7.25 (m, 3H), 2.06 (s, 3H).

^{13}C NMR (101 MHz, $CDCl_3$) 131.5, 128.2, 127.5, 124.0, 85.8, 79.7, 4.2.

(3-Methylbut-1-yn-1-yl)benzene (2j)

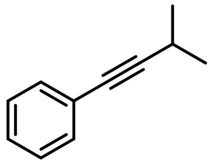

The title compound was synthesized with **1j** using GP-3 on 2.25 mmol scale. The title compound was obtained as colorless oil. *The analytical data matches with the literature precedence.*[17]

R_f: 0.9 (100% pentane)

Yield: 176 mg, 55%

^{1}H NMR (400 MHz, $CDCl_3$) δ 7.44 – 7.36 (m, 2H), 7.32 – 7.22 (m, 3H), 2.77 (h, *J* = 6.9 Hz, 1H), 1.27 (d, *J* = 6.9 Hz, 6H).

^{13}C NMR (101 MHz, $CDCl_3$) δ 131.7, 128.3, 127.6, 124.1, 95.9, 79.8, 23.2, 21.3.

5. Scalability test

We conducted tests at three different scales (1.0 mmol, 2.25 mmol, and 15.0 mmol) to examine the technical applicability. *The results are shown below.*

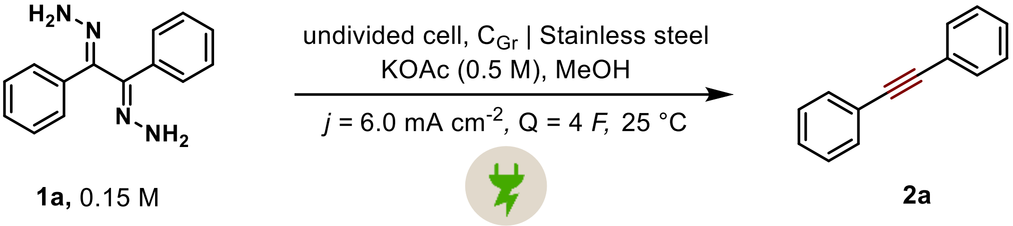

Scale (mmol)	Distance between electrodes (cm)	Stirring rate (rpm)	Time (min)	Applied charge (C)	Yield % (isolated)
1.0	0.5	750	153	385 (4 *F*)	73 (70)
2.25	0.8	750	345	870 (4 *F*)	80 (77)
15.0	0.8	200	2297	5789 (4 *F*)	66 (58)

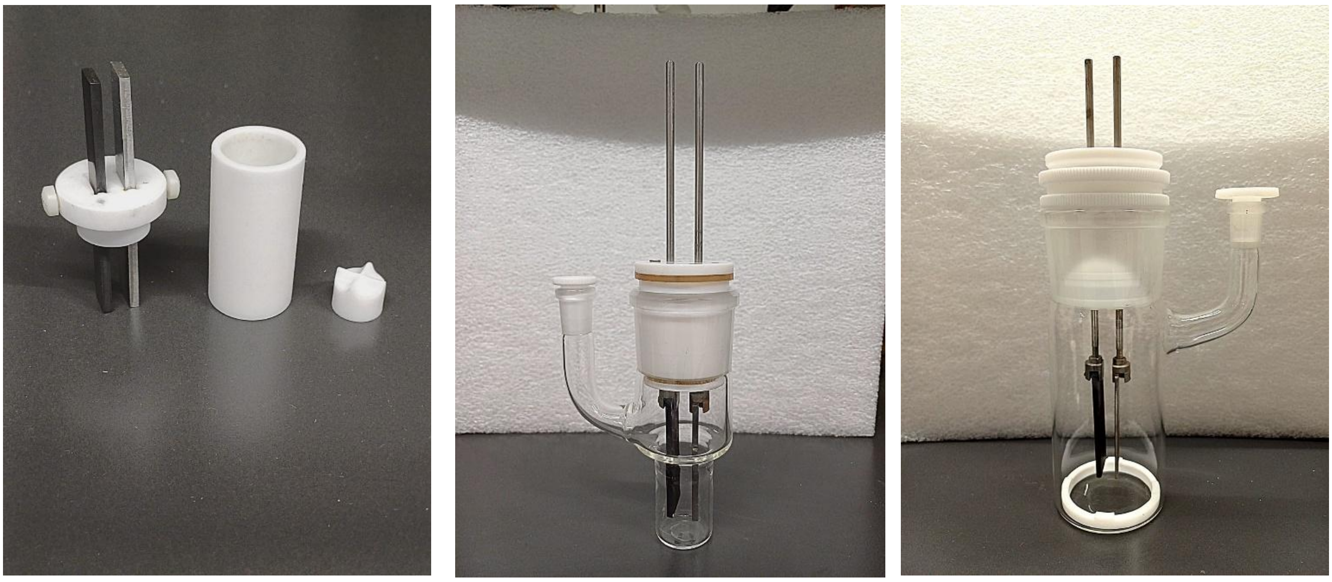

Figure S8. 3 different electrolysis cells for different scale up experiments. 1.0 mmol (*left*; capacity: 7 mL), 2.25 mmol (*middle*; capacity: 25 mL), and 15.0 mmol (*right*; capacity: 100 mL). The diameter of the circular stir bar for 15 mmol scale: 5.8 cm (*inner*), 6.7 cm (*outer*).

6. Reusability test

To assess sustainability[18–20], we evaluated the durability of the electrodes by conducting consecutive electrolysis over eight iterative cycles.

General procedure: The reactions are carried out using the undivided Teflon™ cells with a PTFE lid as described in the section before. Graphite and stainless steel were chosen as the anode and the cathode respectively. The electrodes were cleaned before use. The graphite was rubbed with 1000 grit size sandpaper, whereas the stainless steel was treated with a 0.1 M HCl solution, followed by washing with water and drying.

The glass cell is filled with methanol (7 mL), followed by the addition of KOAc (343 mg, 0.5 M). The reaction mixture was vigorously stirred to ensure maximum solubility. Then **1a** (238 mg, 1.0 mmol, 0.07 M) is added to the reaction mixture and stirred again for solubility aspect. The electrodes were fixed in the PTFE stopper, ensuring the correct height of electrodes dipped for accurate current density (6 mA cm^{-2}). The amperage (42 mA) and amount of applied charge (386 C) are set to the desired values, and the electrolysis was carried out at room temperature under constant stirring. After the required period, 1,3,5-trimethoxybenzene (168 mg) is as added to the reaction mixture as an internal standard. The solution is stirred for 5 min at room temperature to make sure the internal standard is well mixed homogenously. A GC sample is prepared using the reaction mixture and yield is calculated based on the respective area on the GC spectra.

The same electrode setup remained affixed to the lid throughout the procedure. Only the graphite component was cleaned by rinsing with water and acetonitrile to remove the residual salt deposits.

The results are shown below:

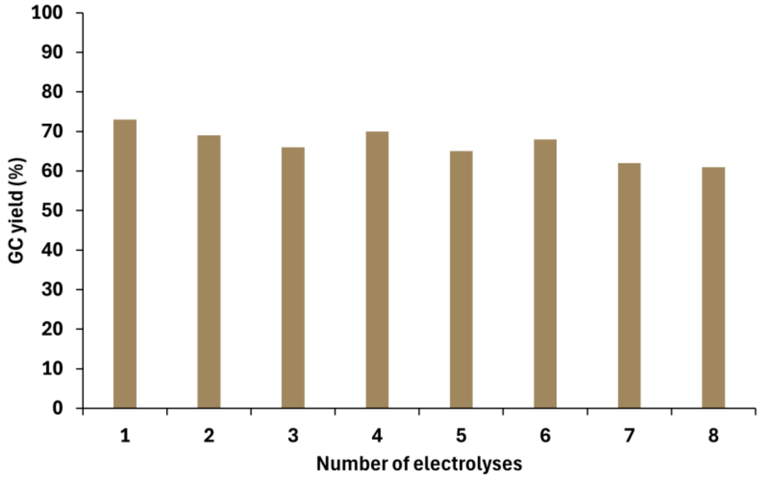

Note: *The cell was washed with water and acetone in between the electrolyses. The electrodes were washed with waster to remove the salt deposited on the electrode.*

7. Cyclic voltammetry Studies

We carried out cyclic voltammetry (CV) studies to understand the multi-oxidation events of hydrazone derivatives (Figure S10). The CVs were measured at 22 °C, starting from the open circuit potential (0 V vs FcH/FcH+) to more anodic potentials (up to +1.75 V vs FcH/FcH+) in the first scan, and the switching potential was +1.75 V vs FcH/FcH+.

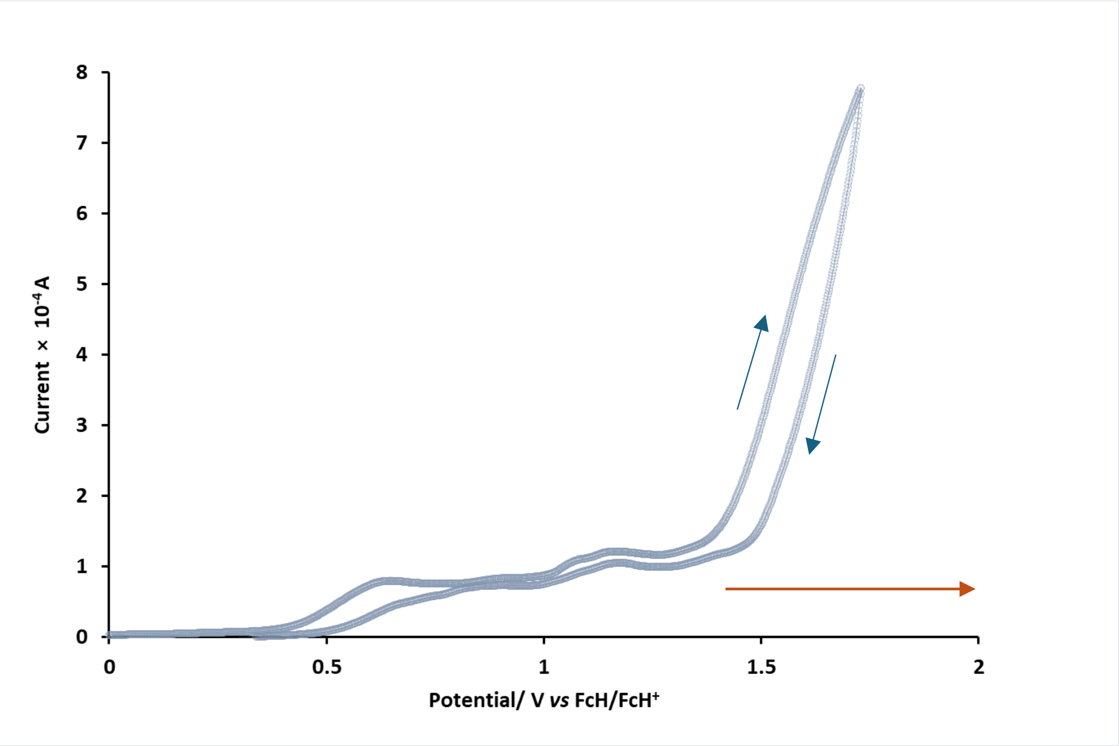

Figure S10: Cyclic voltammogram (IUPAC) of 10 mM **1a** with 0.1 M NBu_4PF_6 as supporting electrolyte. Ag^+/Ag reference electrode (RE), glassy carbon as working electrode (WE) and counter electrode (CE). Scan rate: 100 mV/s; Temperature: 22 °C; Start point: 0 V; Switching potential: 1.75 V; End point: 0 V; Direction: Oxidative (shown in brown arrow).

Conclusion: Two oxidation peaks are observed in cyclic voltammetry measurements. One corresponds to the oxidation of first N–H bond ($E_{vs\ FcH/FcH+}$ = 0.66 V) and the second corresponds to the oxidation of resulting diazene derivative after initial oxidation ($E_{vs\ FcH/FcH+}$ = 1.15 V).

8. Spectra

^{1}H NMR spectrum (400 MHz, CD_2Cl_2) of **1a**.

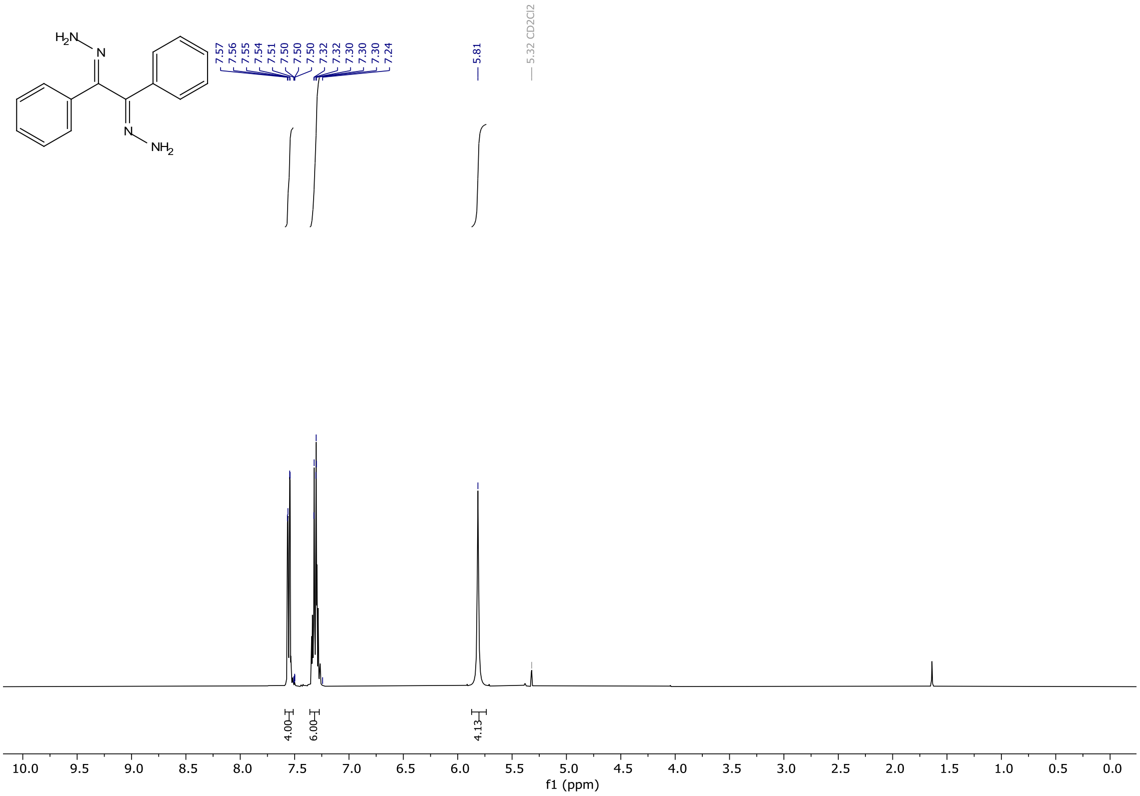

^{13}C NMR spectrum (101 MHz, CD_2Cl_2) of **1a**.

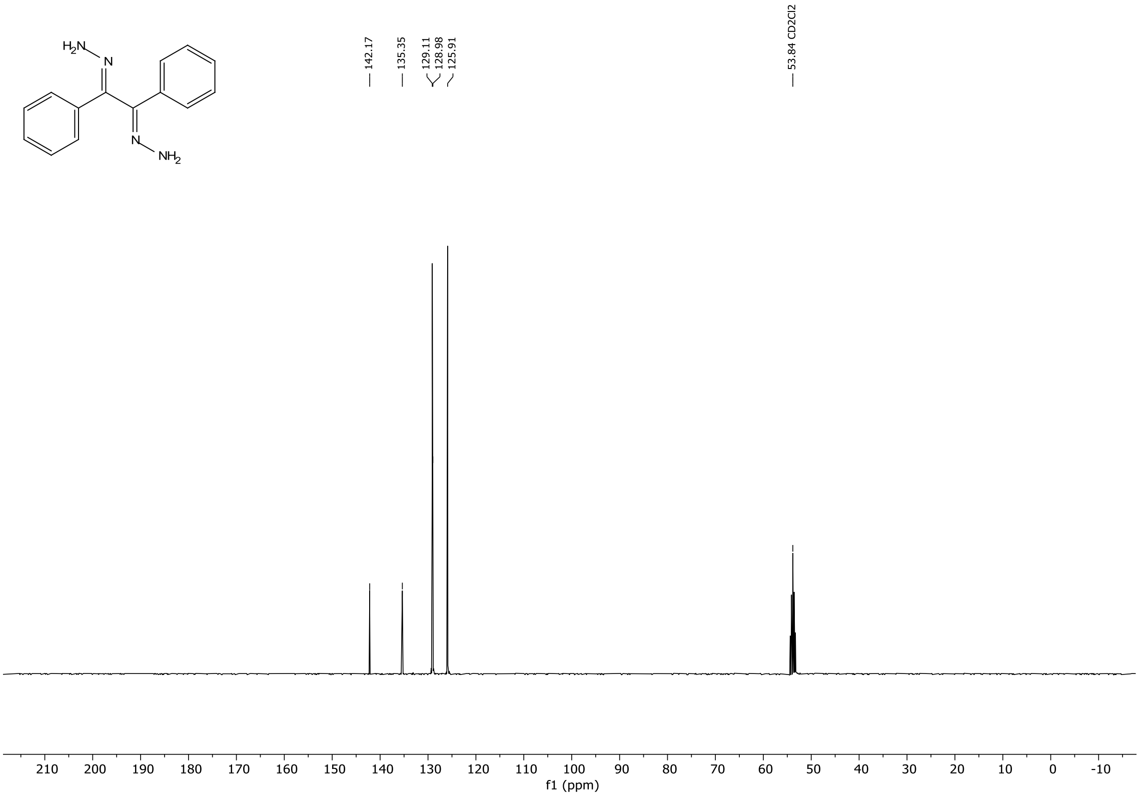

^{1}H NMR spectrum (400 MHz, CD_2Cl_2) of **1b**.

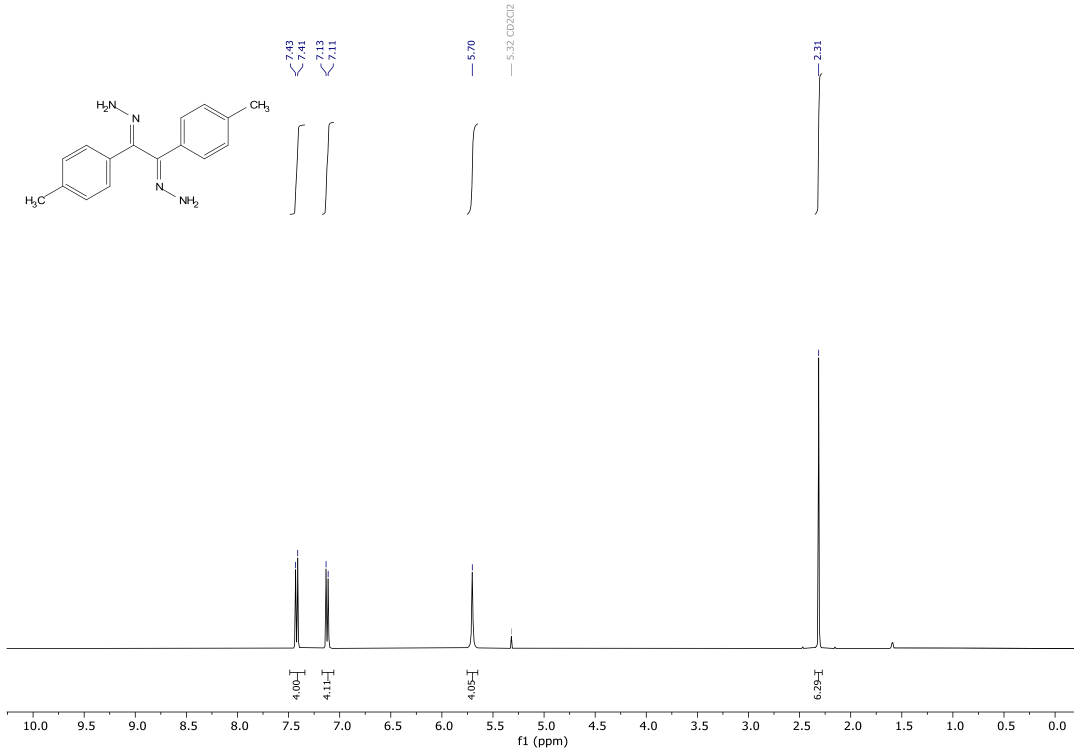

^{13}C NMR spectrum (101 MHz, CD_2Cl_2) of **1b**.

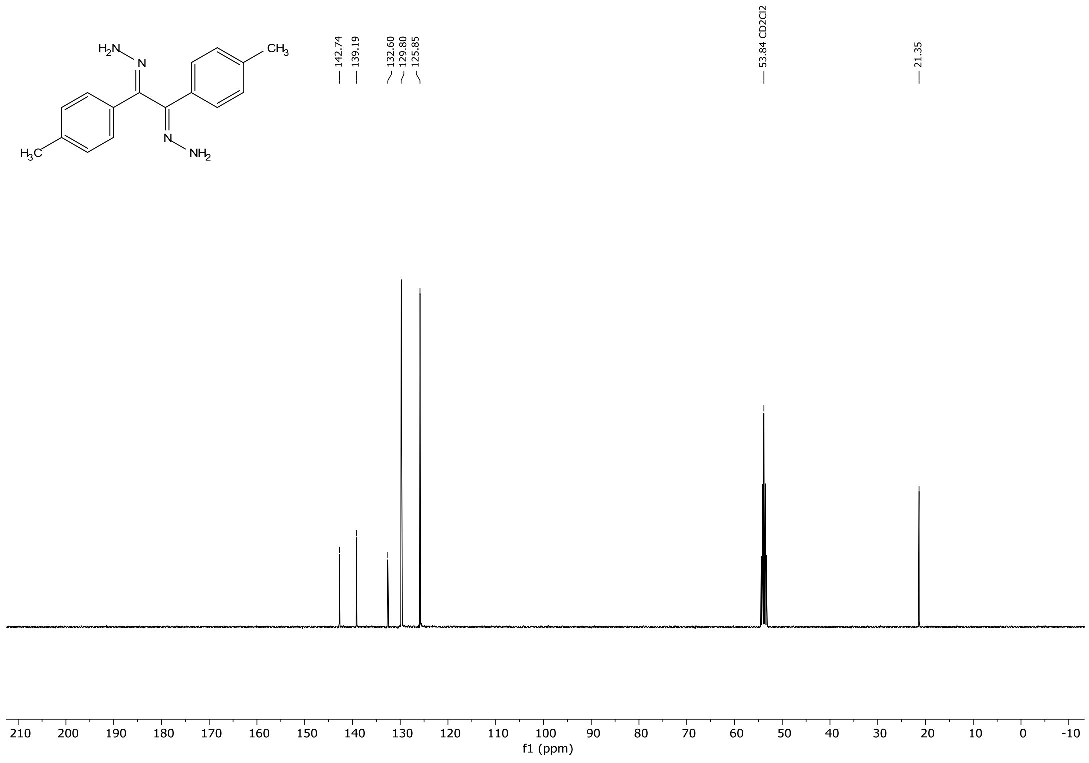

^{1}H NMR spectrum (400 MHz, $CDCl_3$) of **1c**.

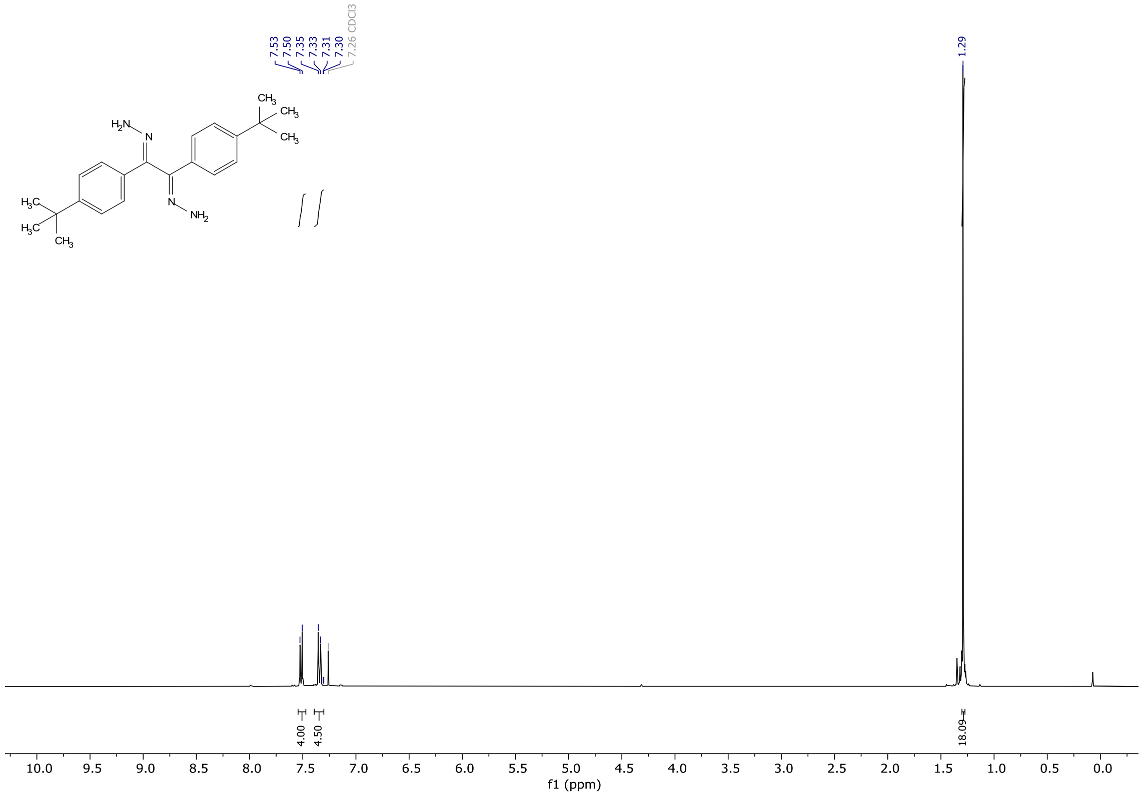

^{13}C NMR spectrum (101 MHz, $CDCl_3$) of **1c**.

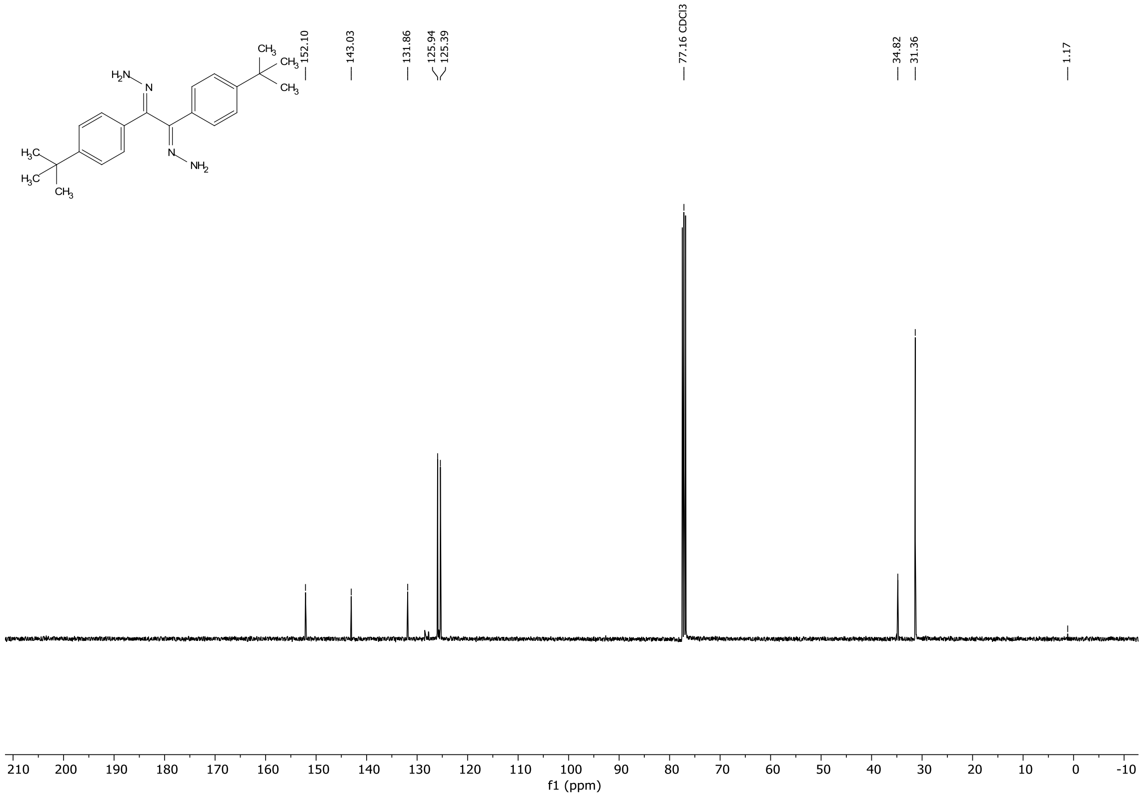

^{1}H NMR spectrum (400 MHz, CD_2Cl_2) of **1d**.

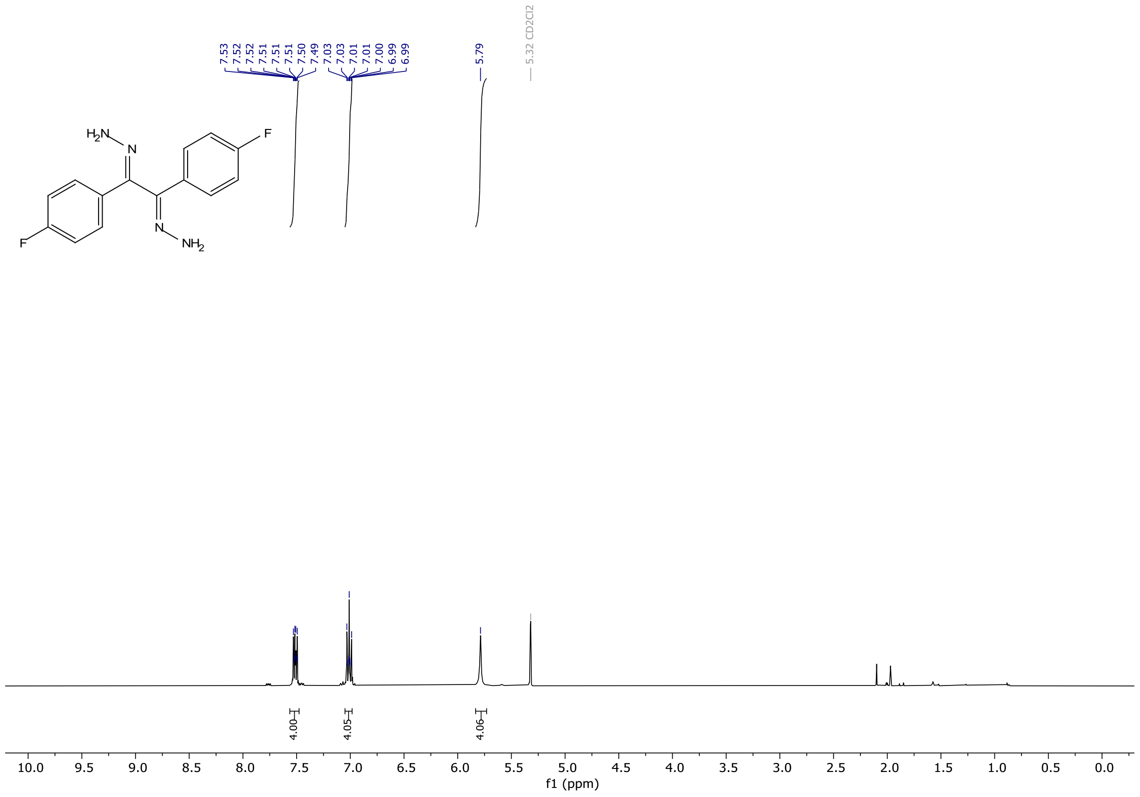

^{13}C NMR spectrum (101 MHz, CD_2Cl_2) of **1d**.

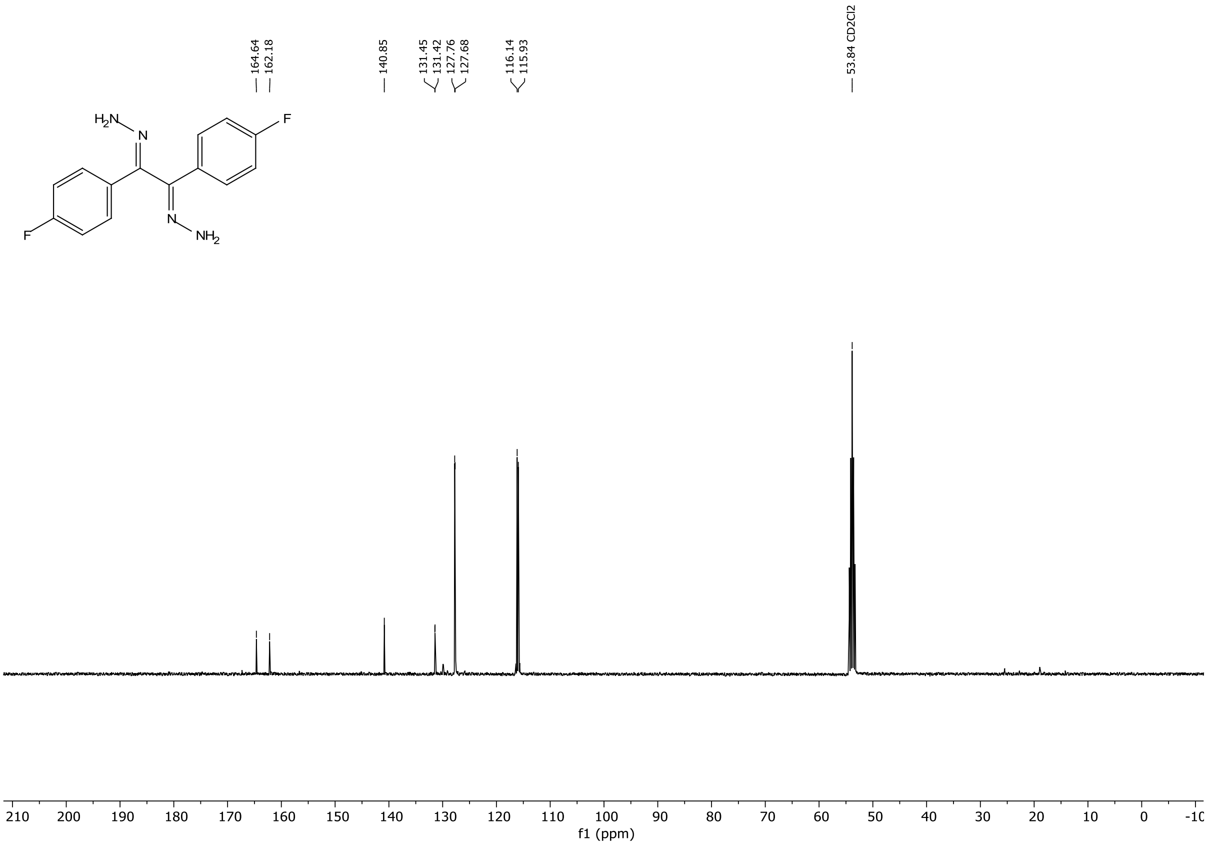

^{19}F NMR spectrum (376 MHz, CD_2Cl_2) of **1d**.

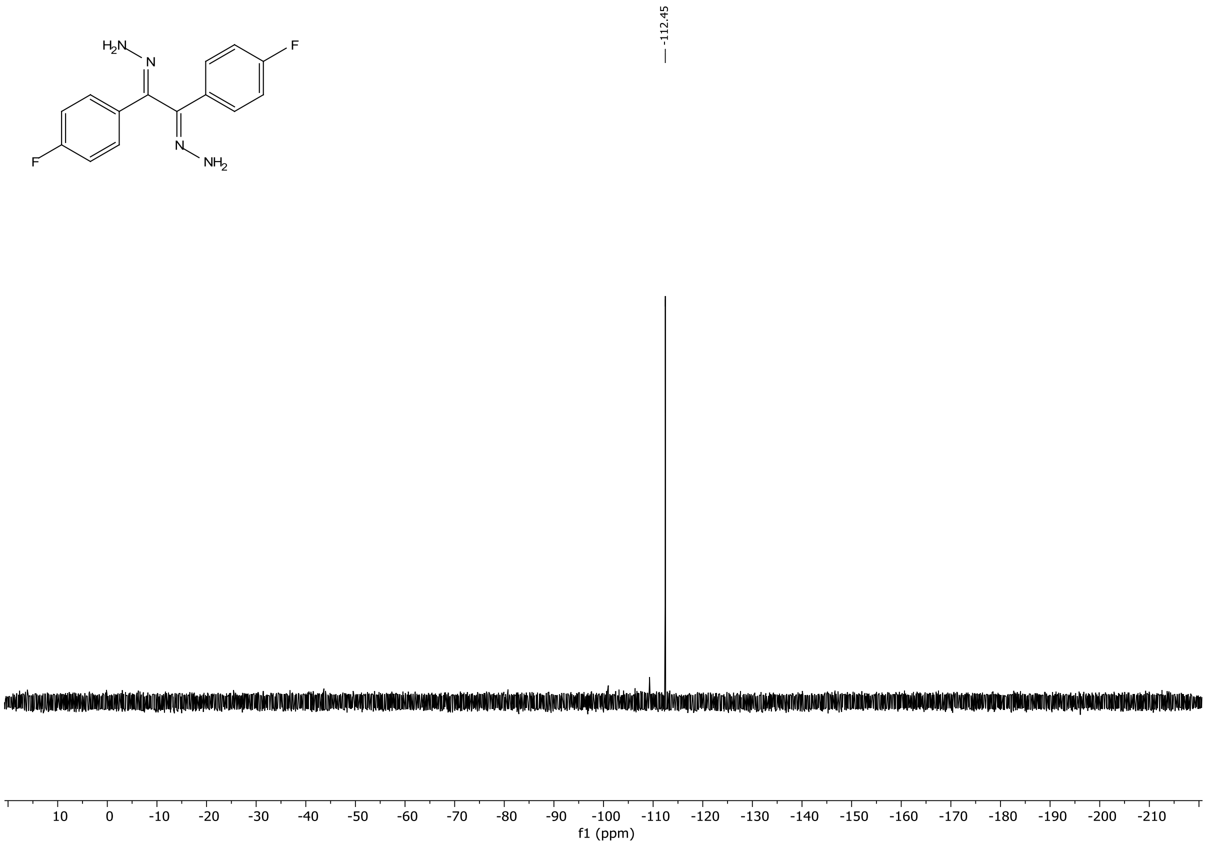

^{1}H NMR spectrum (400 MHz, CD_2Cl_2) of **1e**.

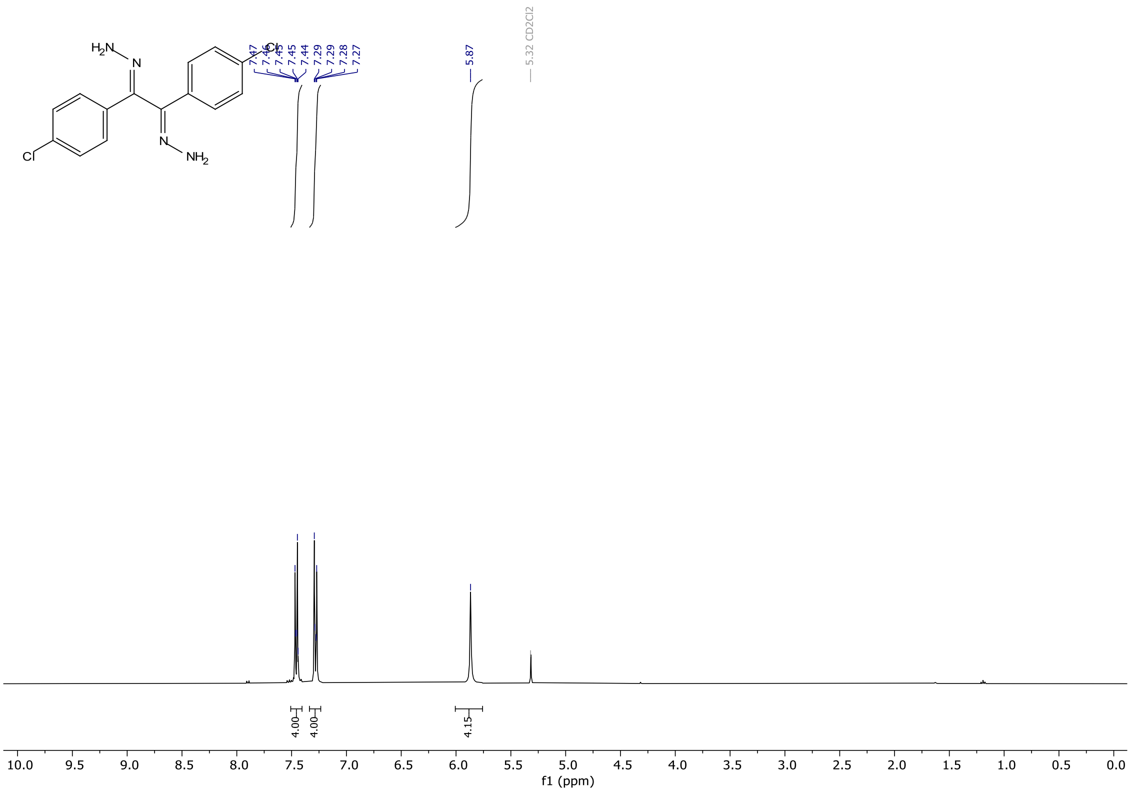

^{13}C NMR spectrum (101 MHz, CD_2Cl_2) of **1e**.

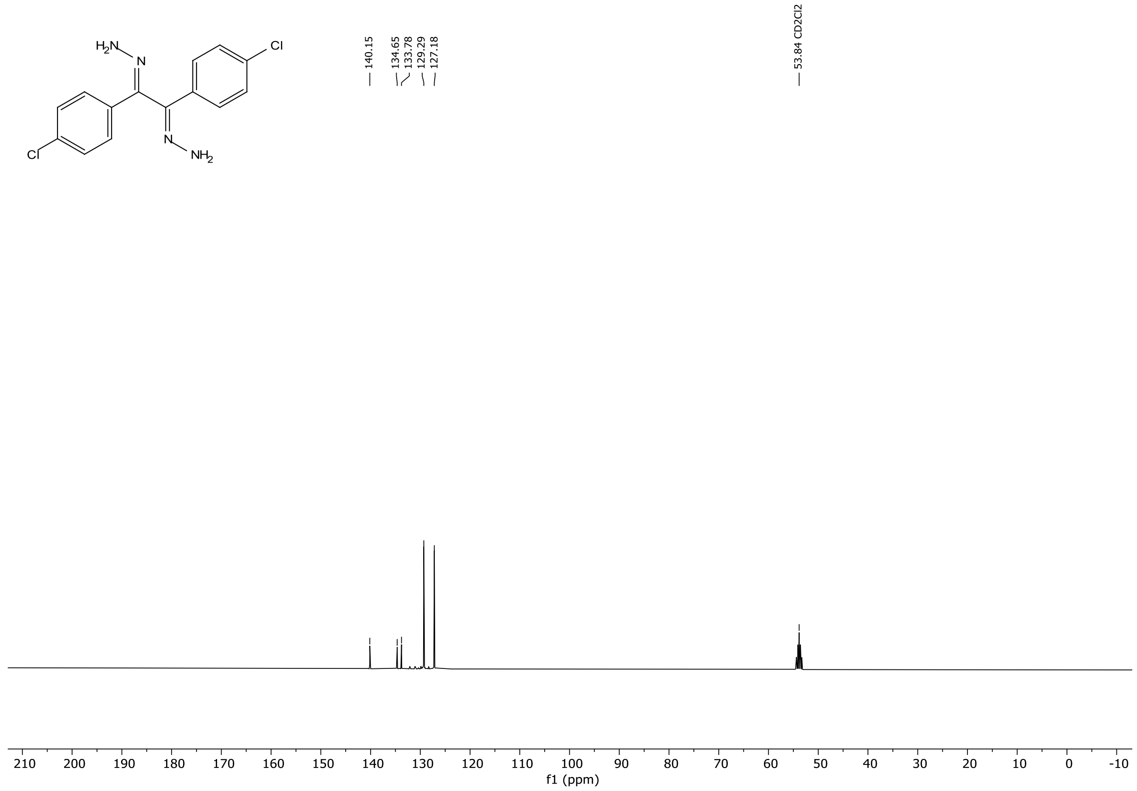

^{1}H NMR spectrum (400 MHz, CD_2Cl_2) of **1f**.

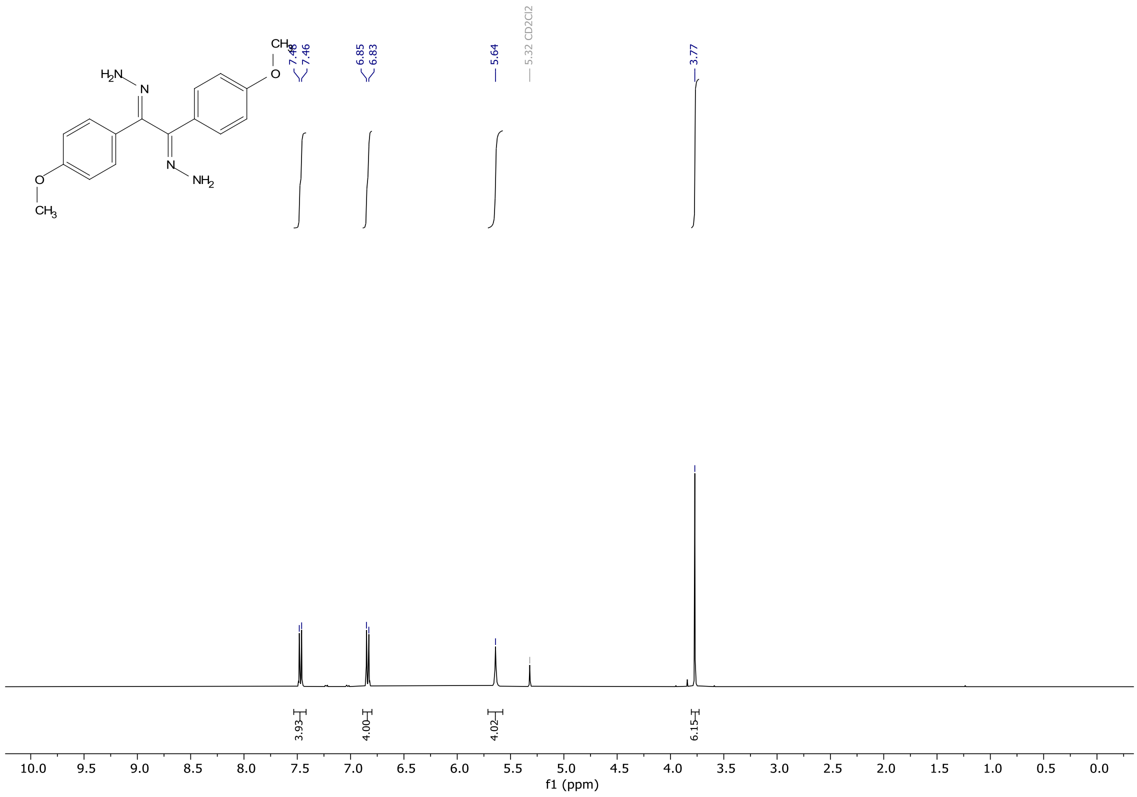

^{13}C NMR spectrum (101 MHz, CD_2Cl_2) of **1f**.

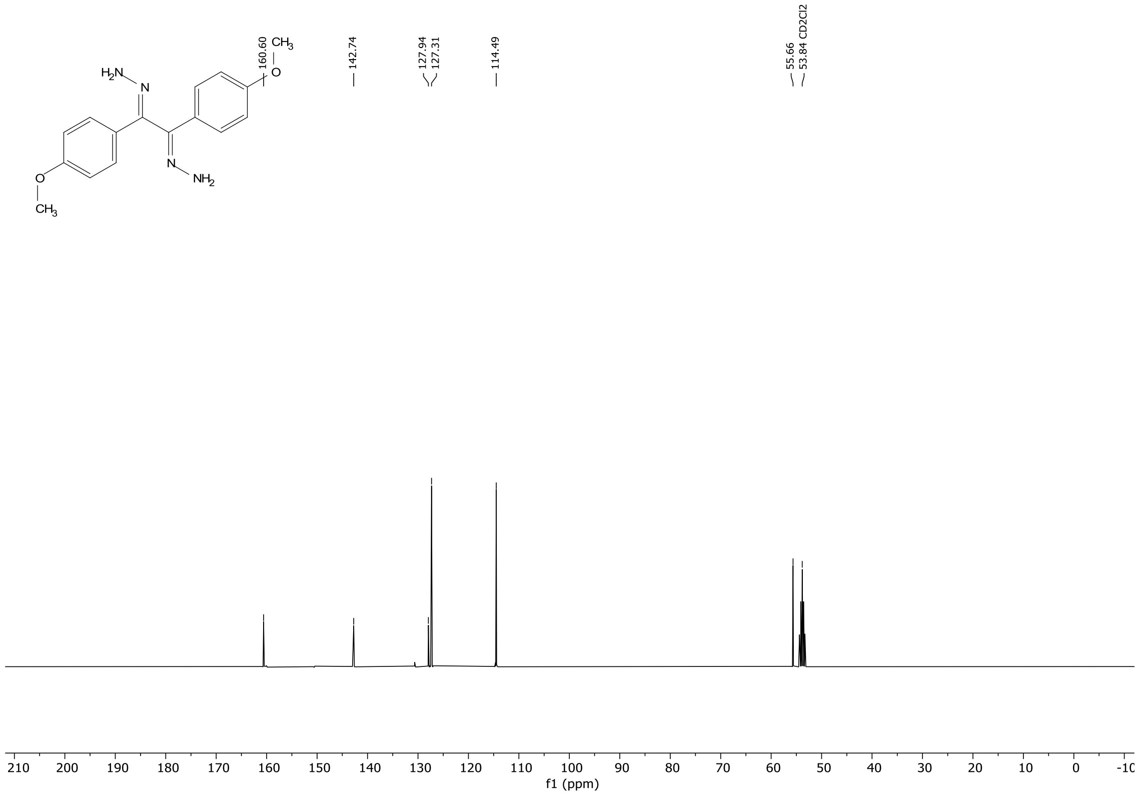

^{1}H NMR spectrum (400 MHz, $CDCl_3$) of **1g**.

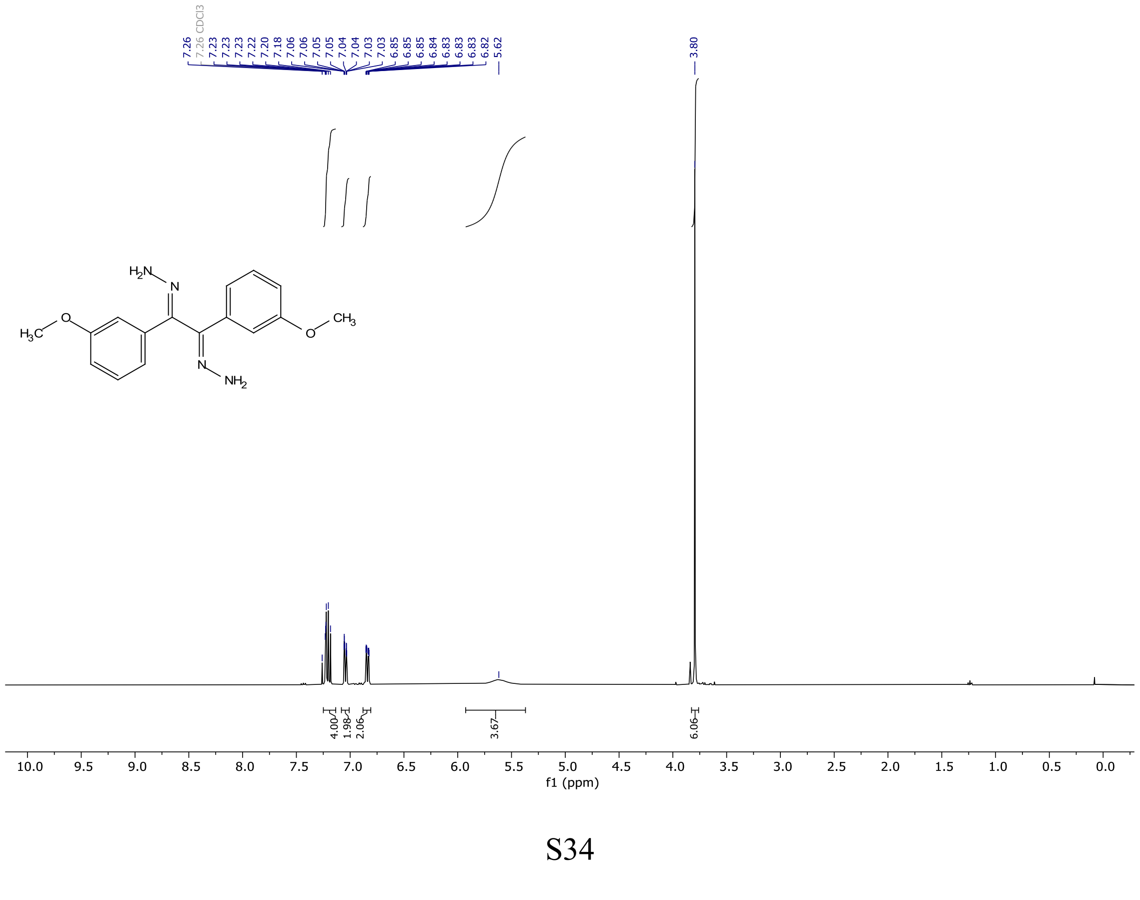

^{13}C NMR spectrum (101 MHz, $CDCl_3$) of **1g**.

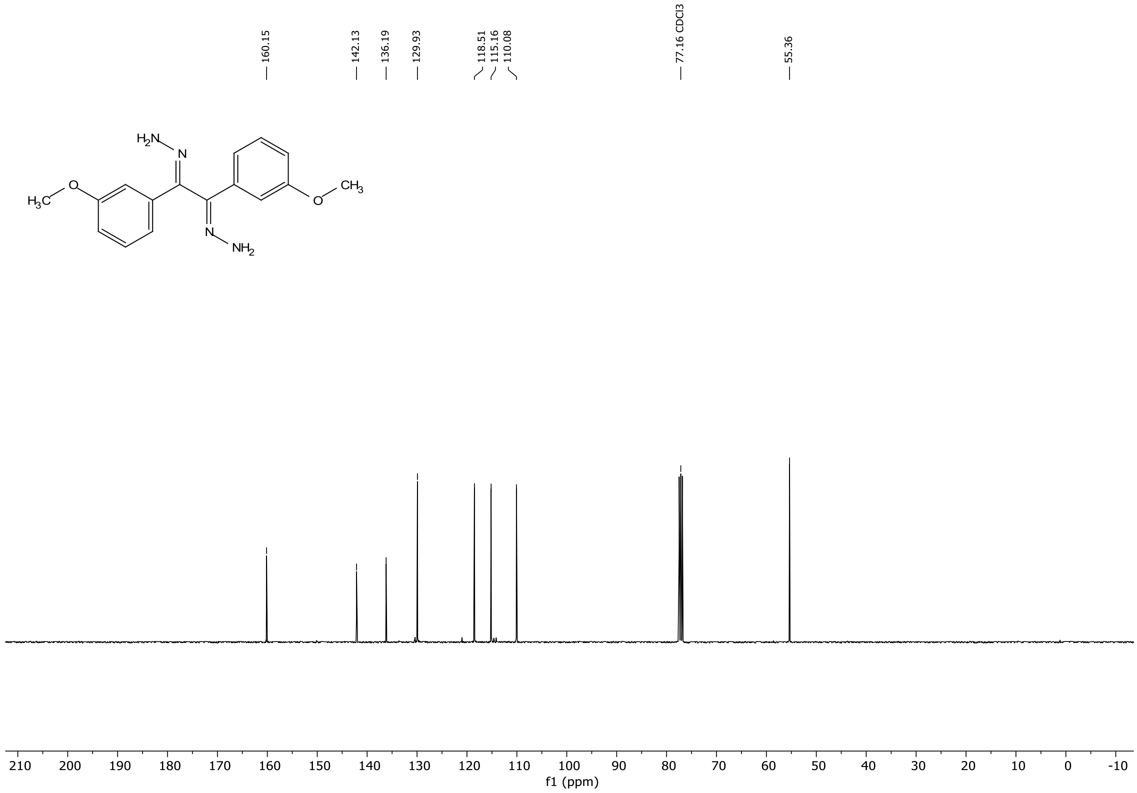

^{1}H NMR spectrum (400 MHz, CD_2Cl_2) of **1h**.

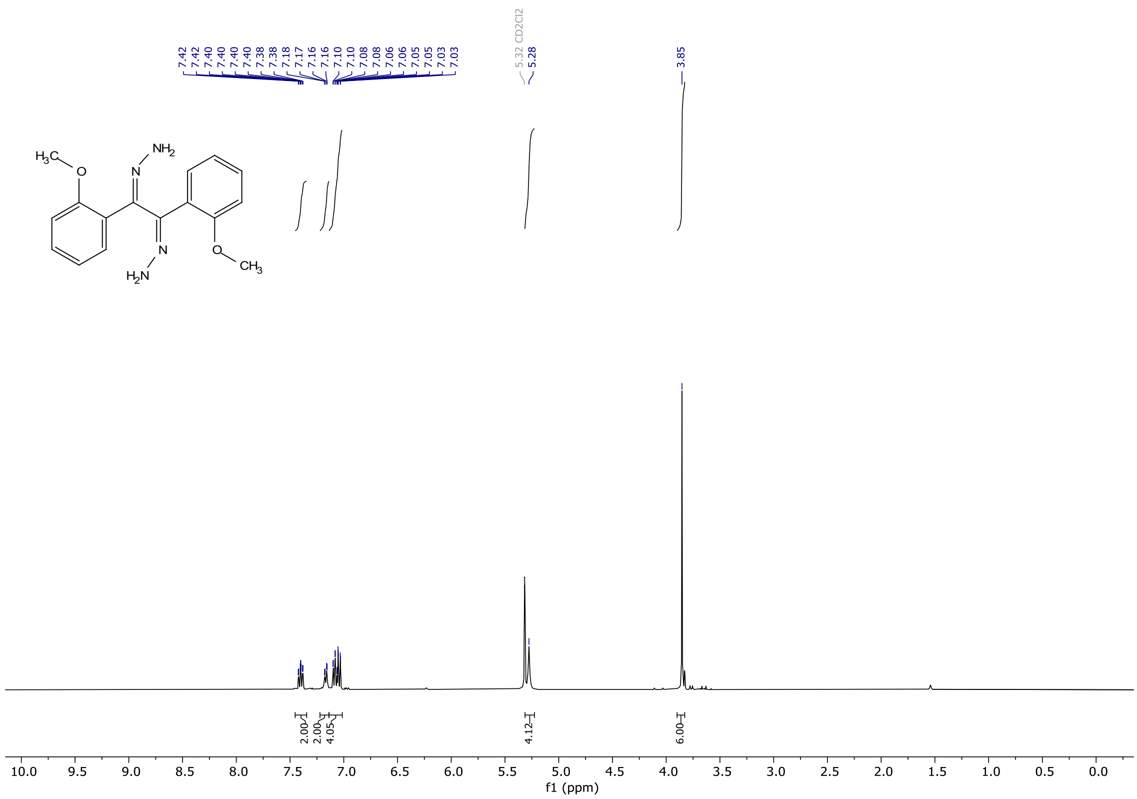

^{13}C NMR spectrum (101 MHz, CD_2Cl_2) of **1h**.

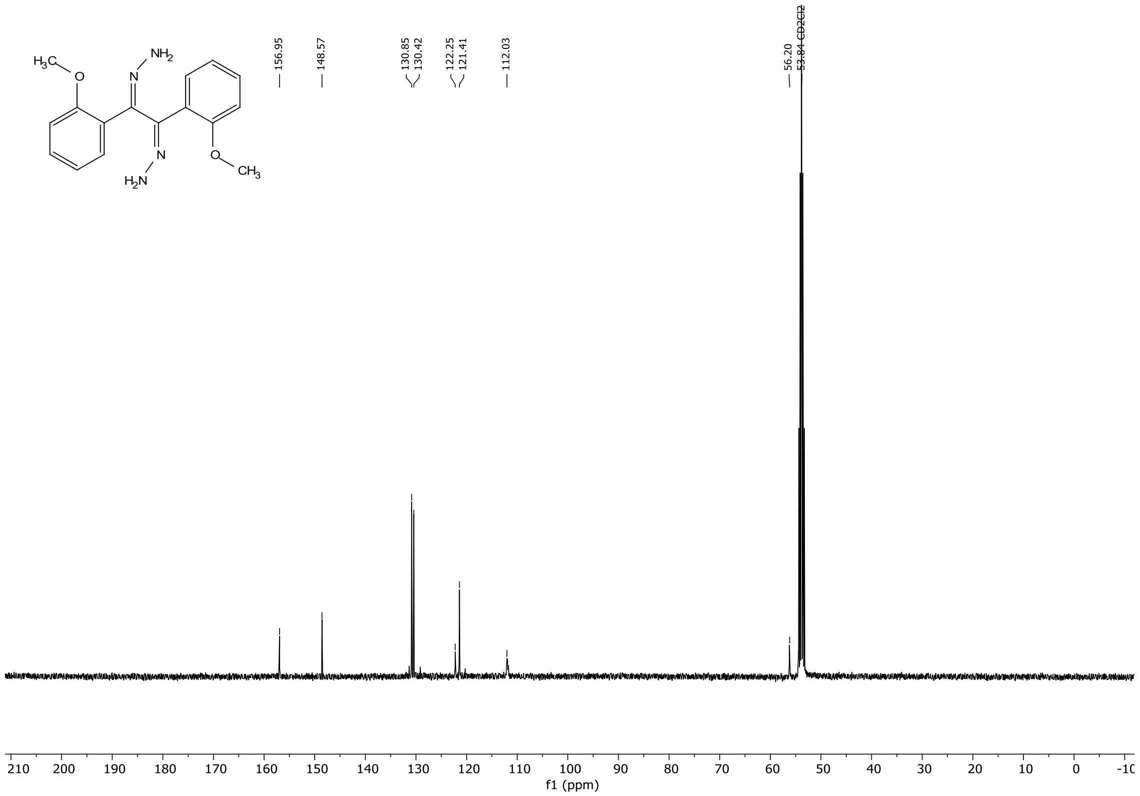

^{1}H NMR spectrum (400 MHz, CD_2Cl_2) of **1i**.

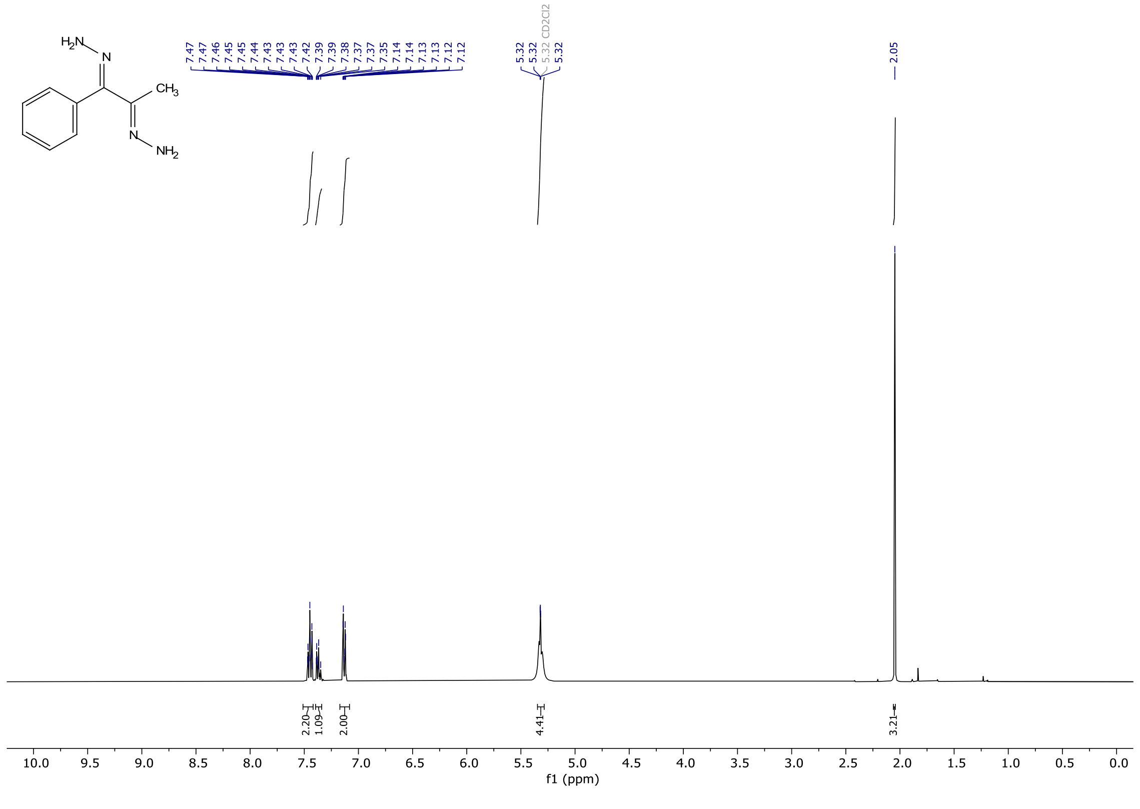

^{13}C NMR spectrum (101 MHz, CD_2Cl_2) of **1i**.

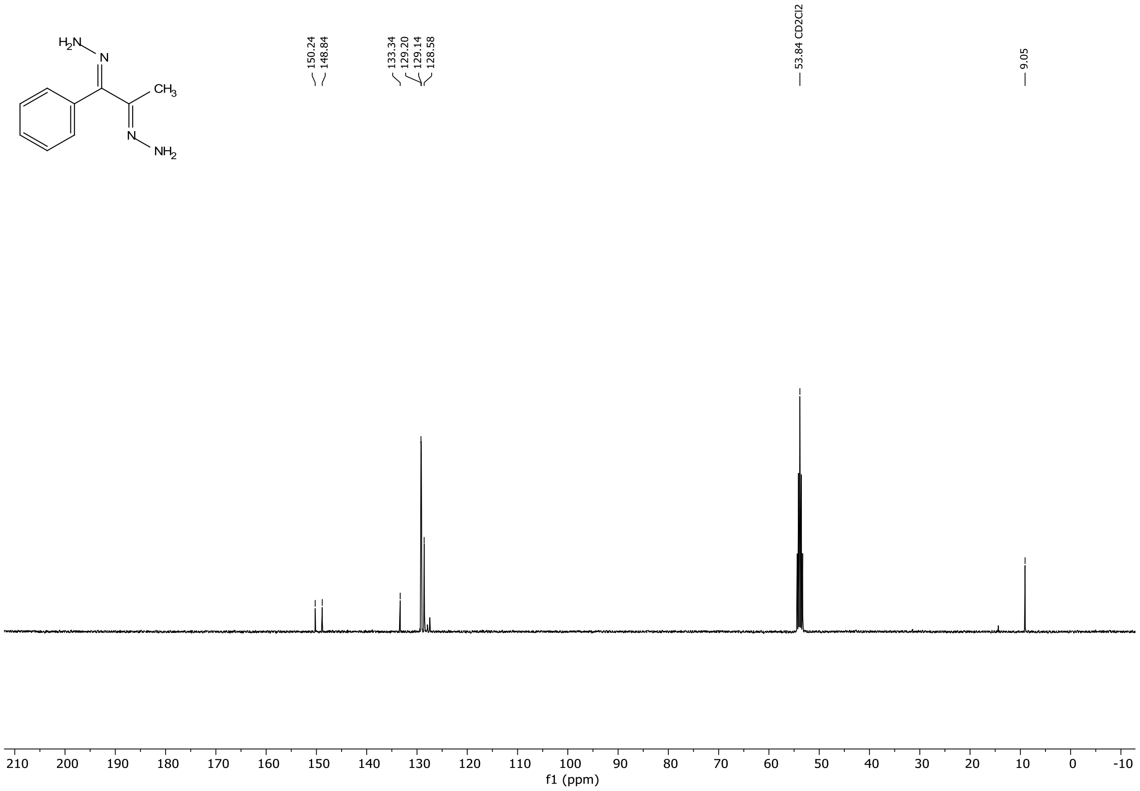

^{1}H NMR spectrum (400 MHz, CD_2Cl_2) of **1j**.

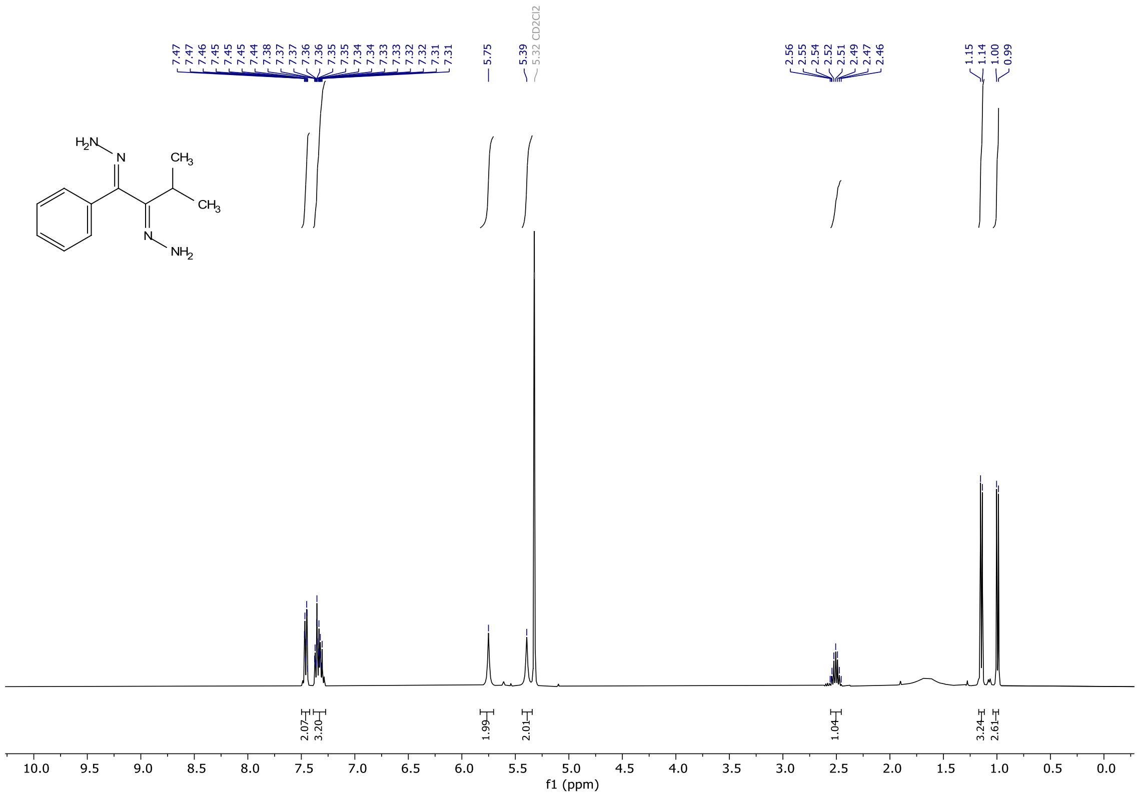

^{13}C NMR spectrum (101 MHz, CD_2Cl_2) of **1j**.

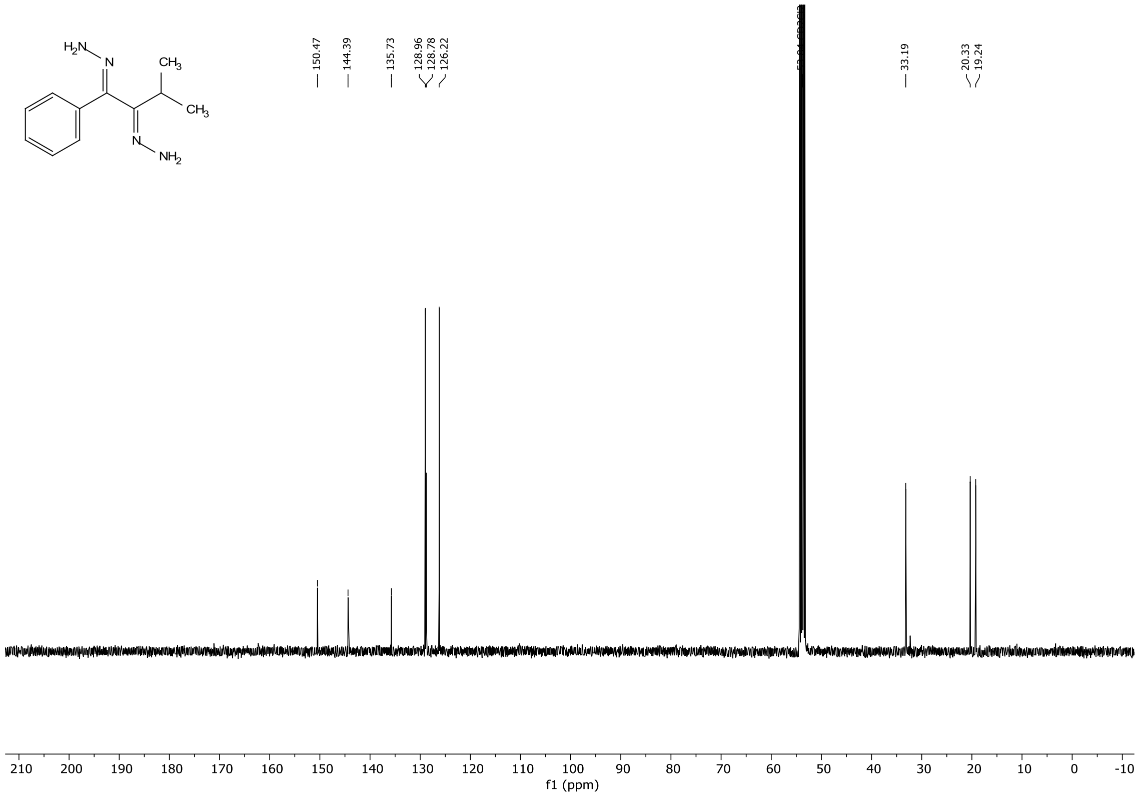

^{1}H NMR spectrum (400 MHz, CD_2Cl_2) of **1k**.

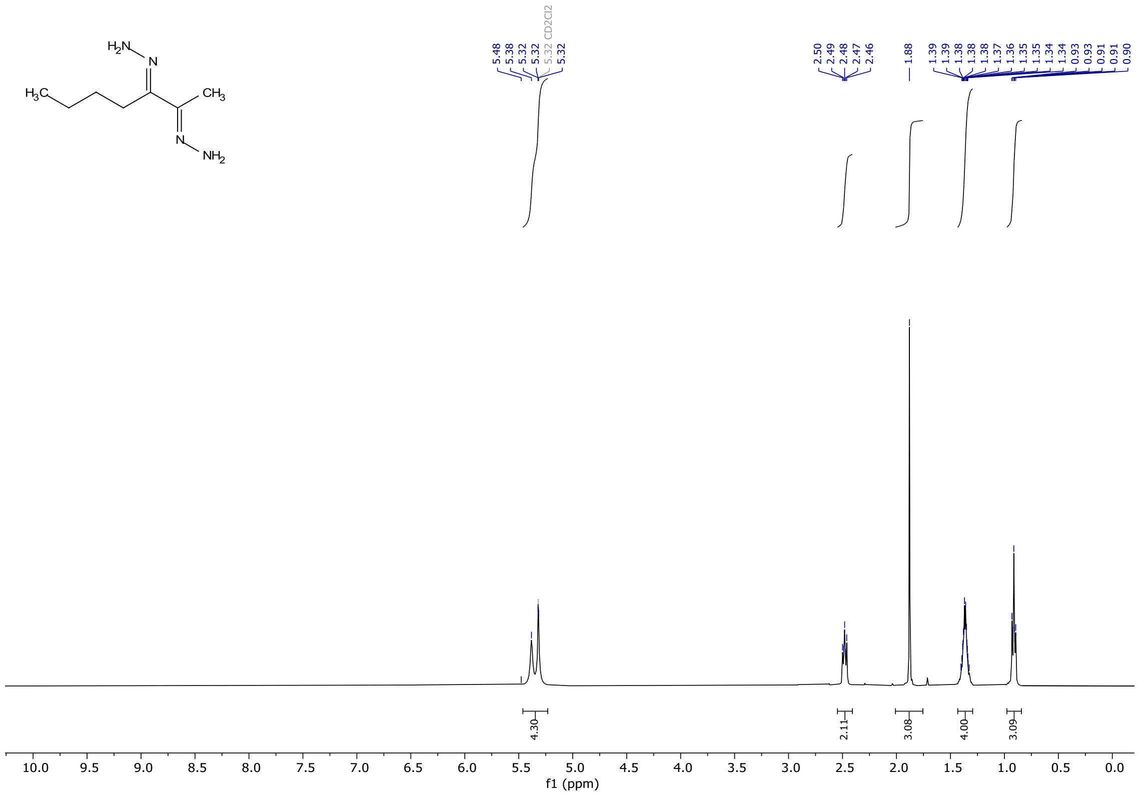

^{13}C NMR spectrum (101 MHz, CD_2Cl_2) of **1k**.

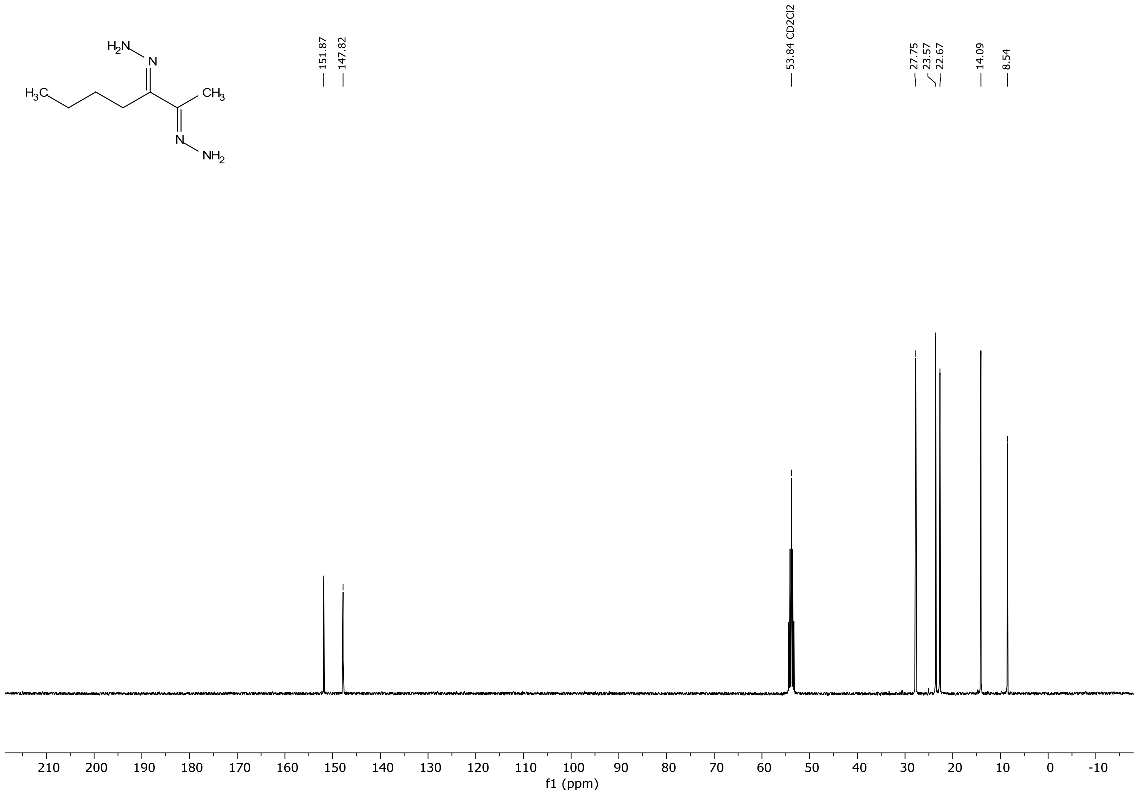

^{1}H NMR spectrum (400 MHz, $CDCl_3$) of **2a**.

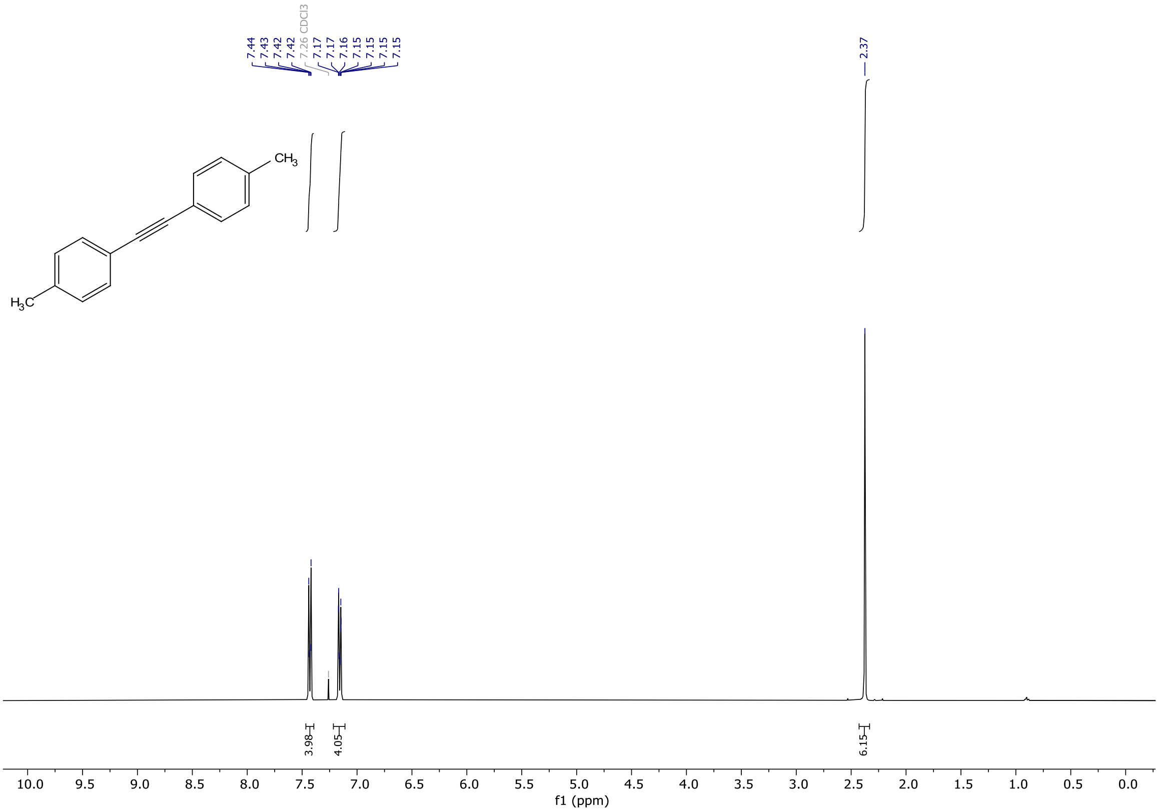

^{13}C NMR spectrum (101 MHz, $CDCl_3$) of **2a**.

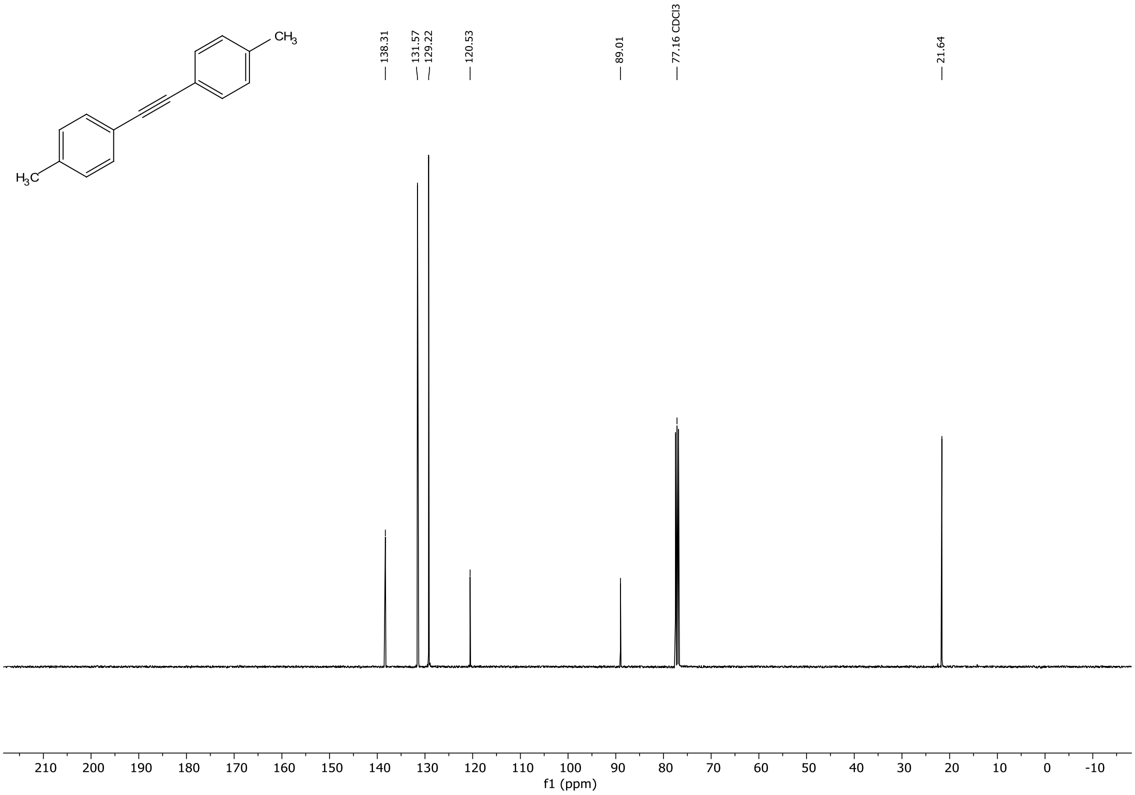

^{1}H NMR spectrum (400 MHz, $CDCl_3$) of **2c**.

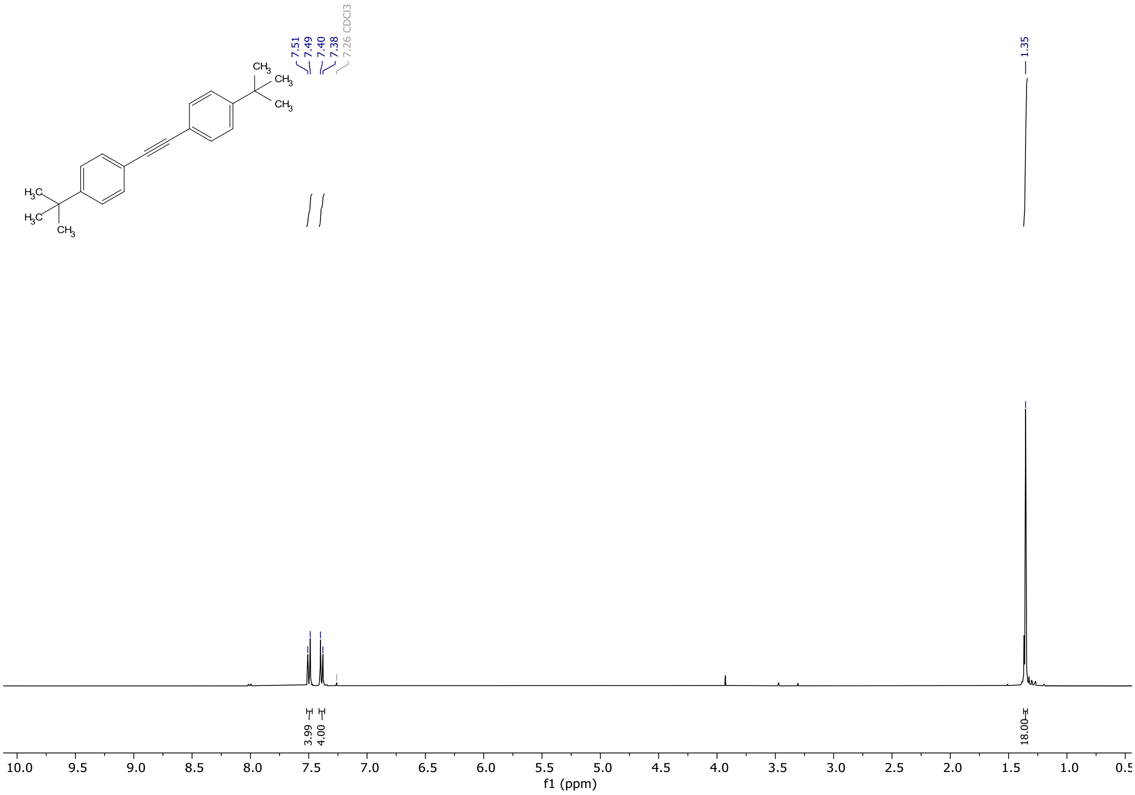

^{13}C NMR spectrum (101 MHz, $CDCl_3$) of **2c**.

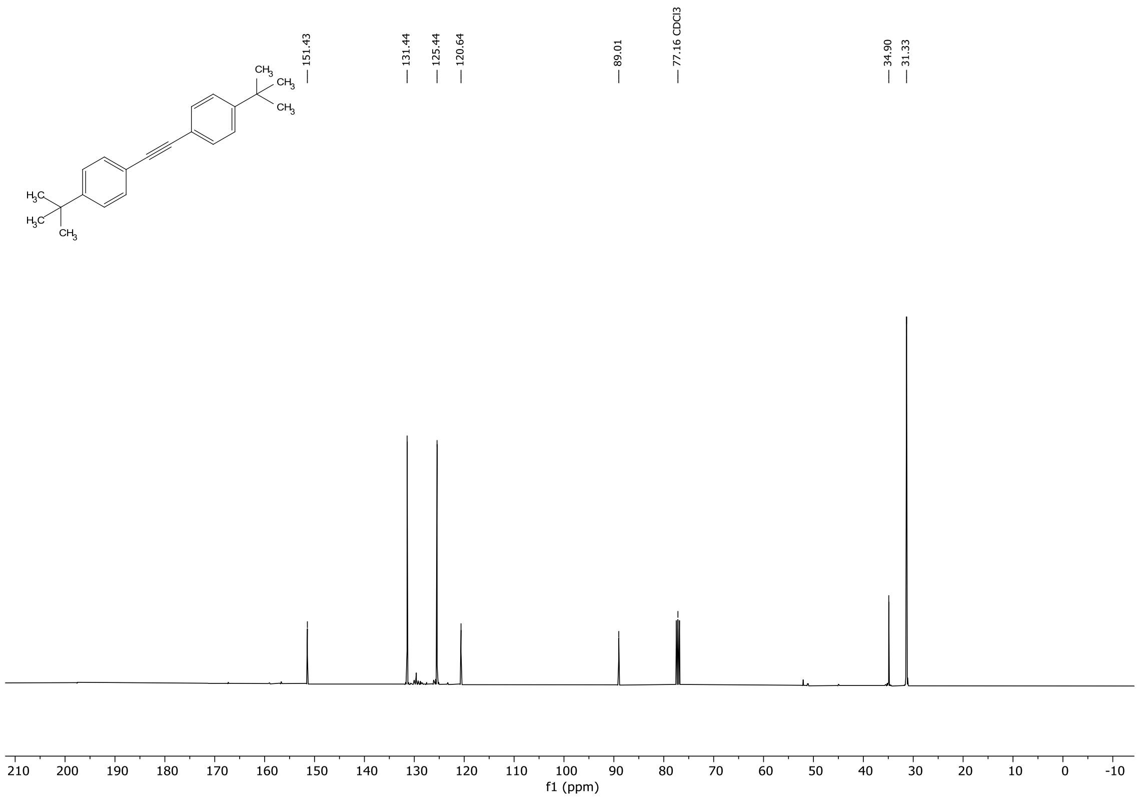

^{1}H NMR spectrum (400 MHz, $CDCl_3$) of **2e**.

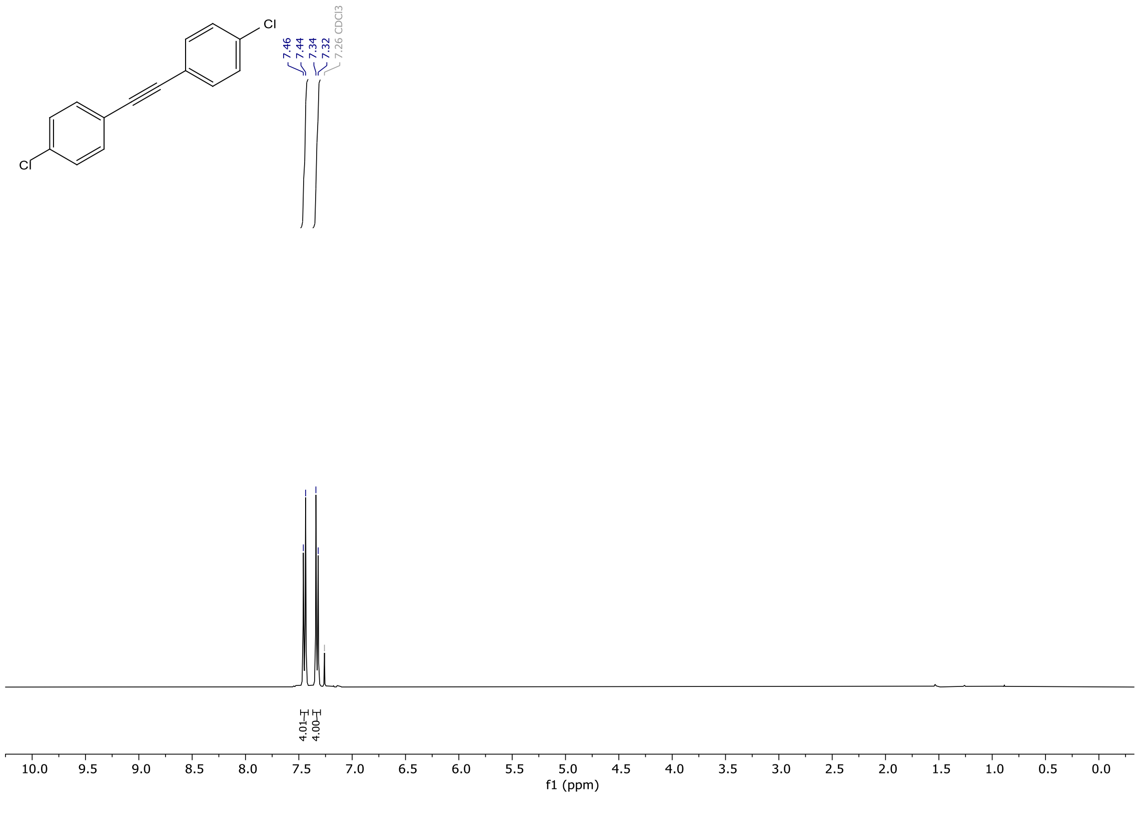

^{13}C NMR spectrum (101 MHz, $CDCl_3$) of **2e**.

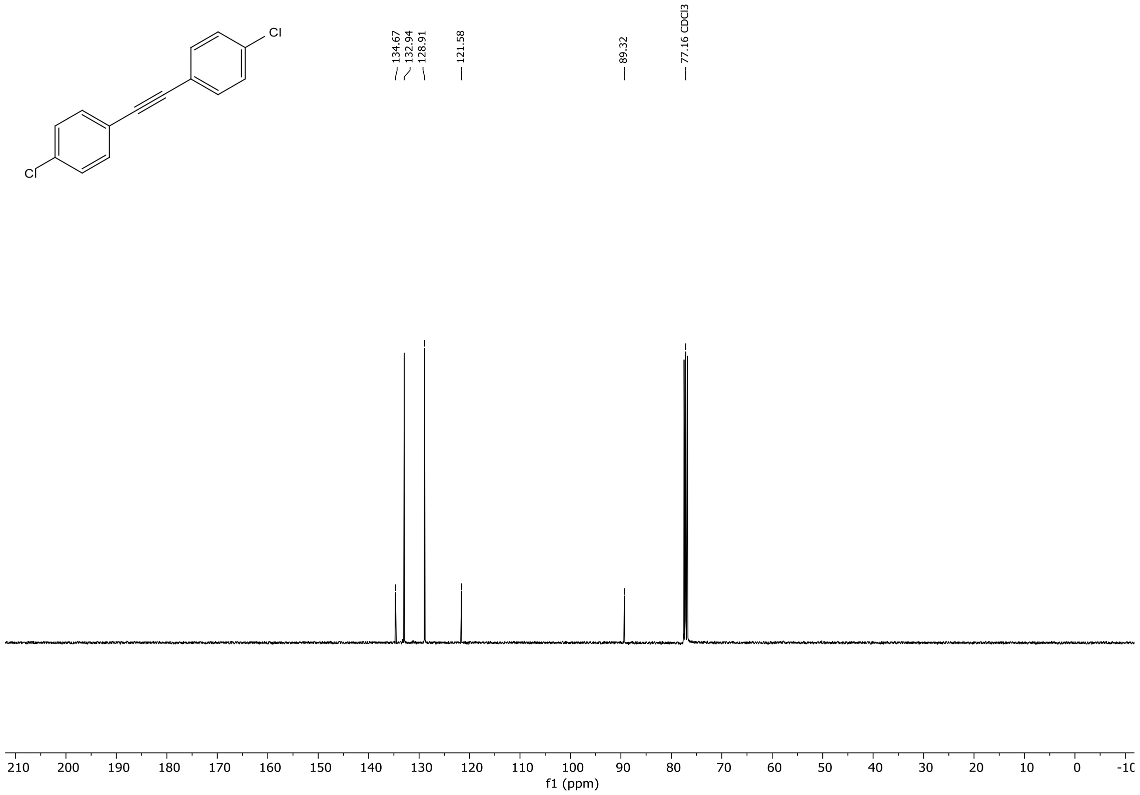

^{1}H NMR spectrum (400 MHz, $CDCl_3$) of **2f**.

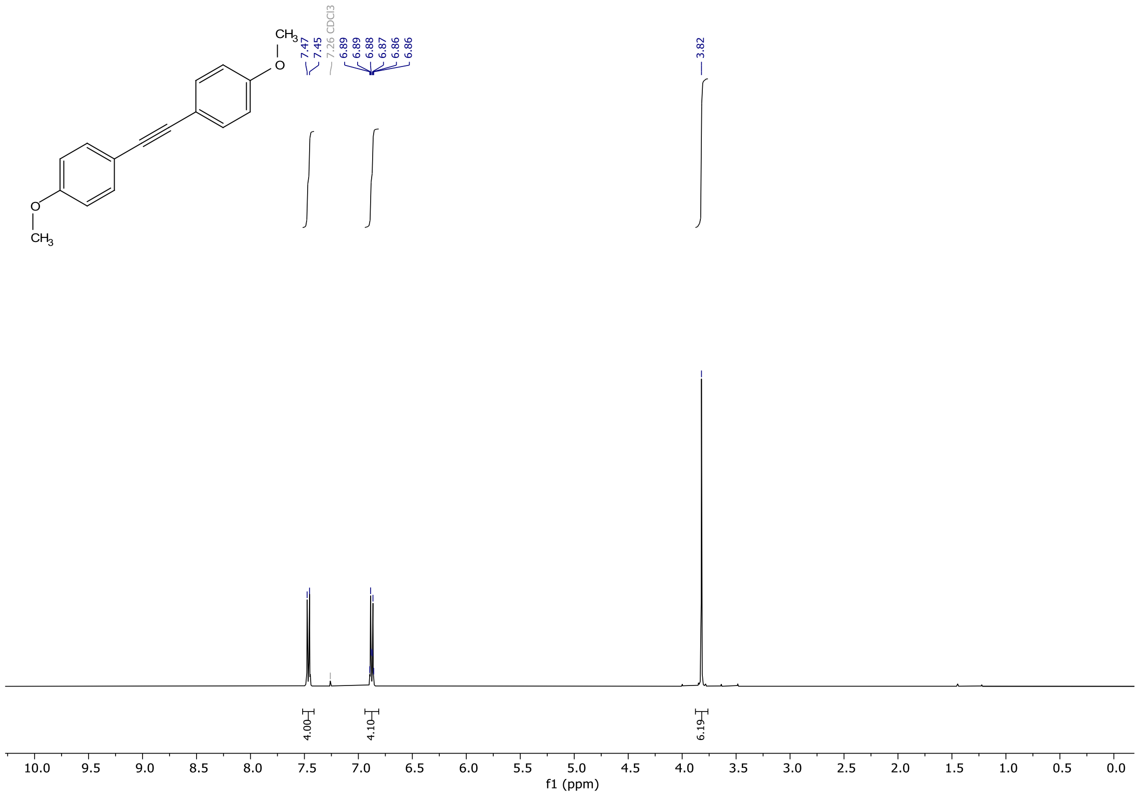

^{13}C NMR spectrum (101 MHz, $CDCl_3$) of **2f**.

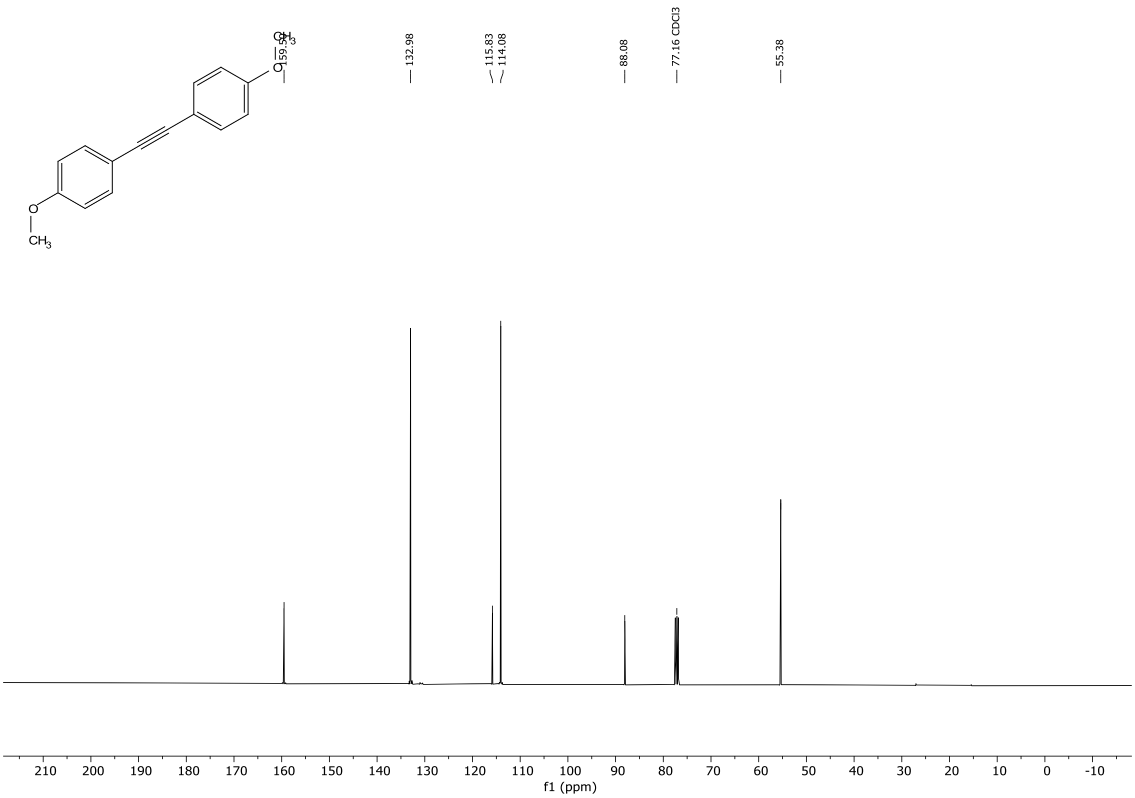

^{1}H NMR spectrum (400 MHz, $CDCl_3$) of **2g**.

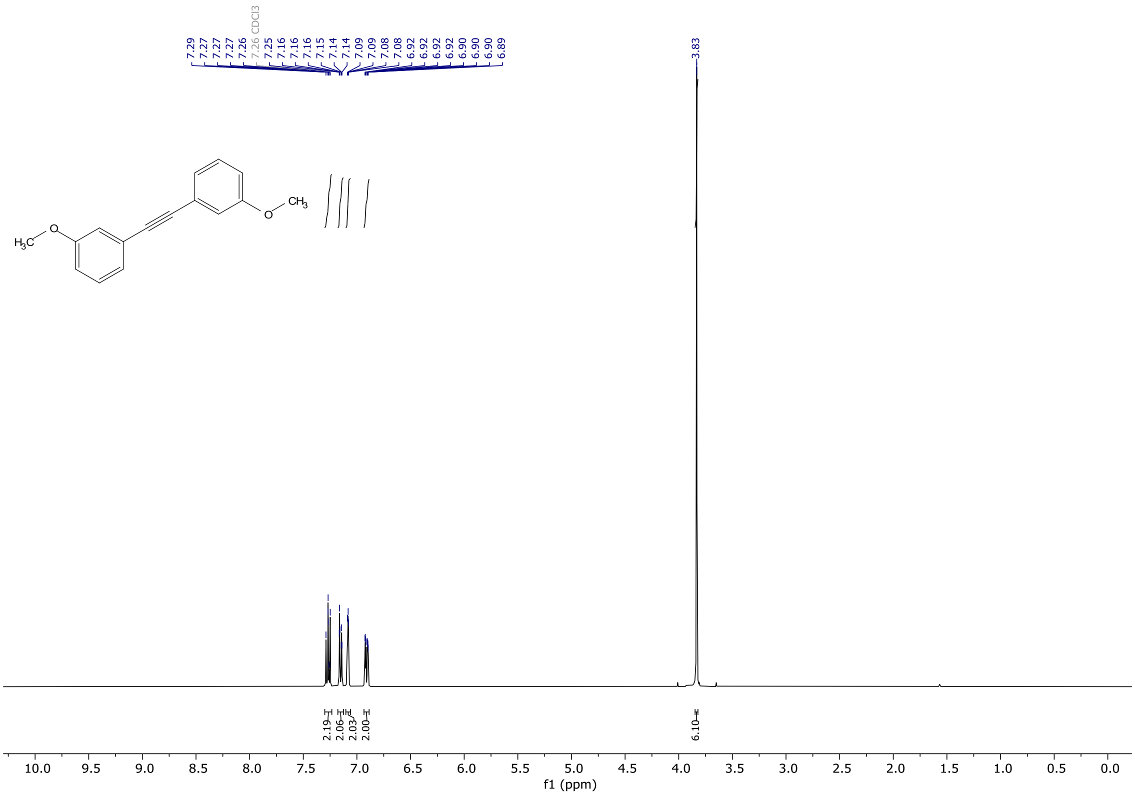

^{13}C NMR spectrum (101 MHz, $CDCl_3$) of **2g**.

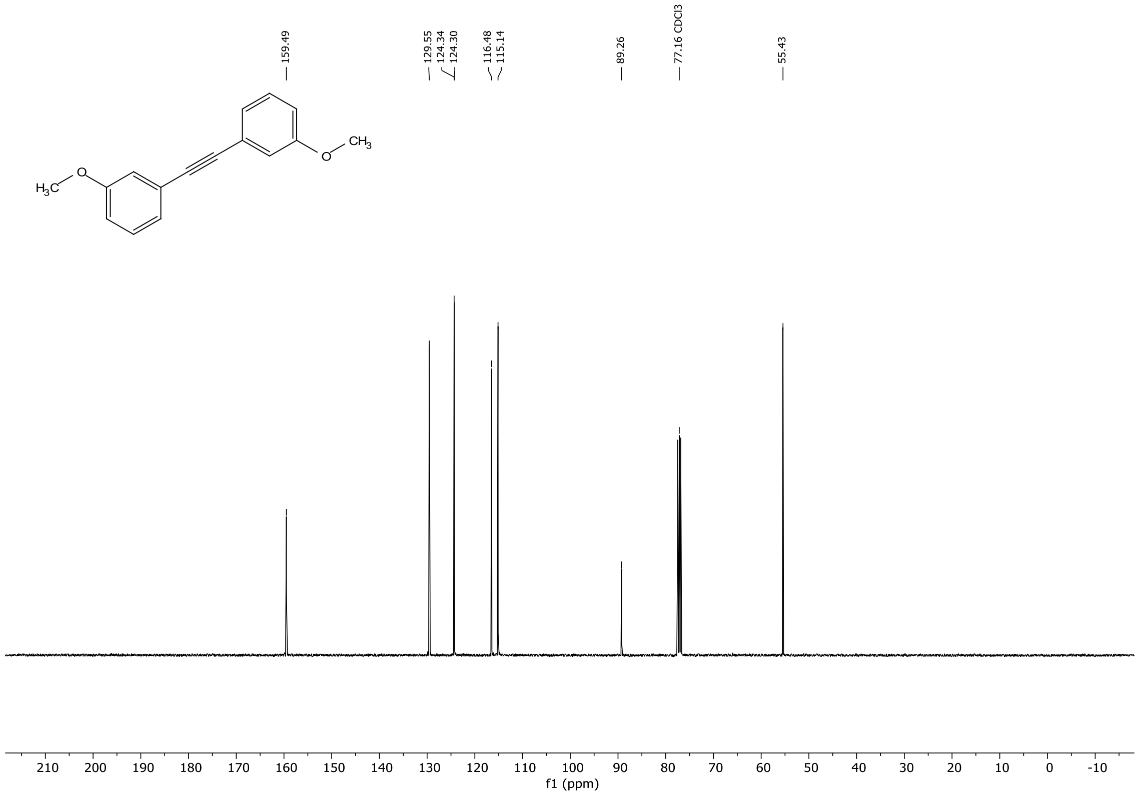

^{1}H NMR spectrum (400 MHz, $CDCl_3$) of **2h**.

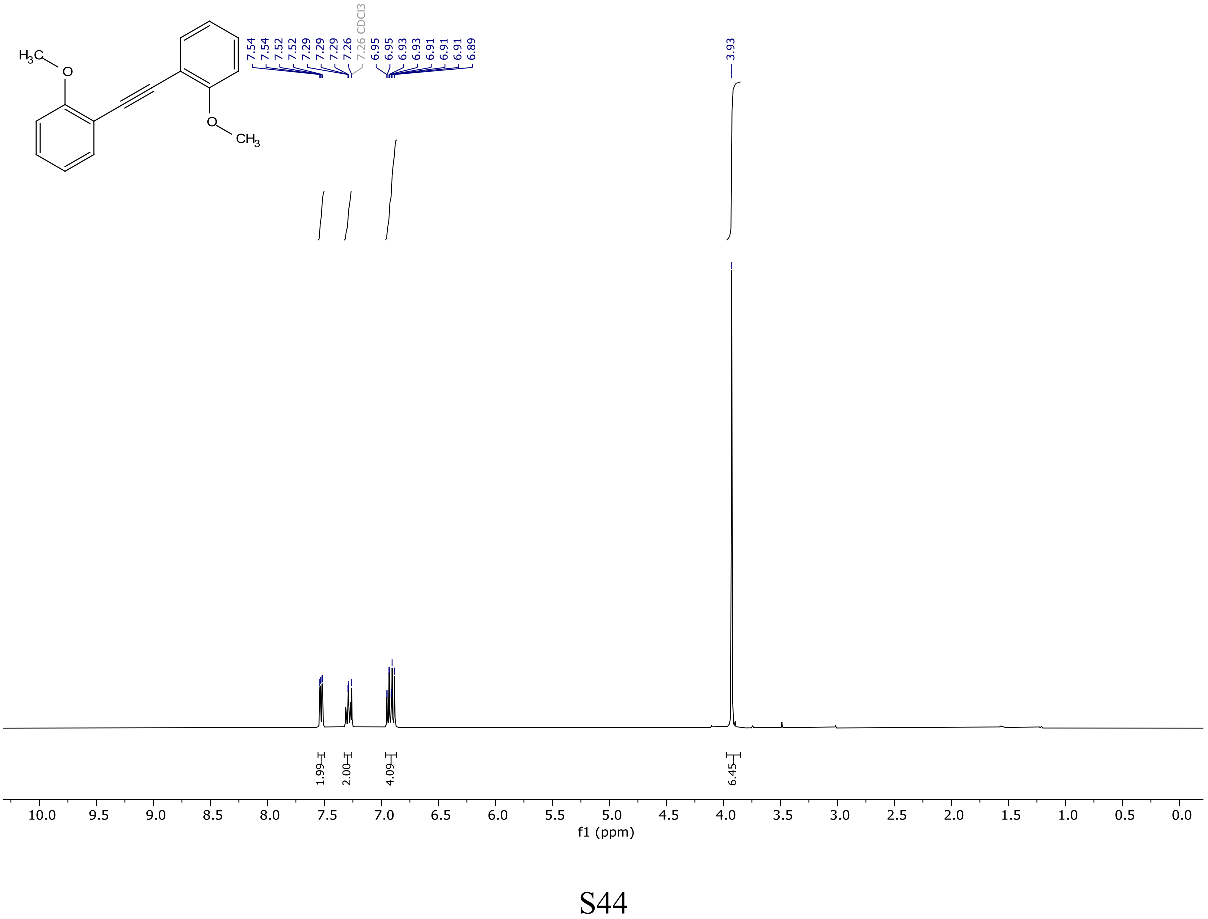

^{13}C NMR spectrum (101 MHz, CD_2Cl_2) of **2h**.

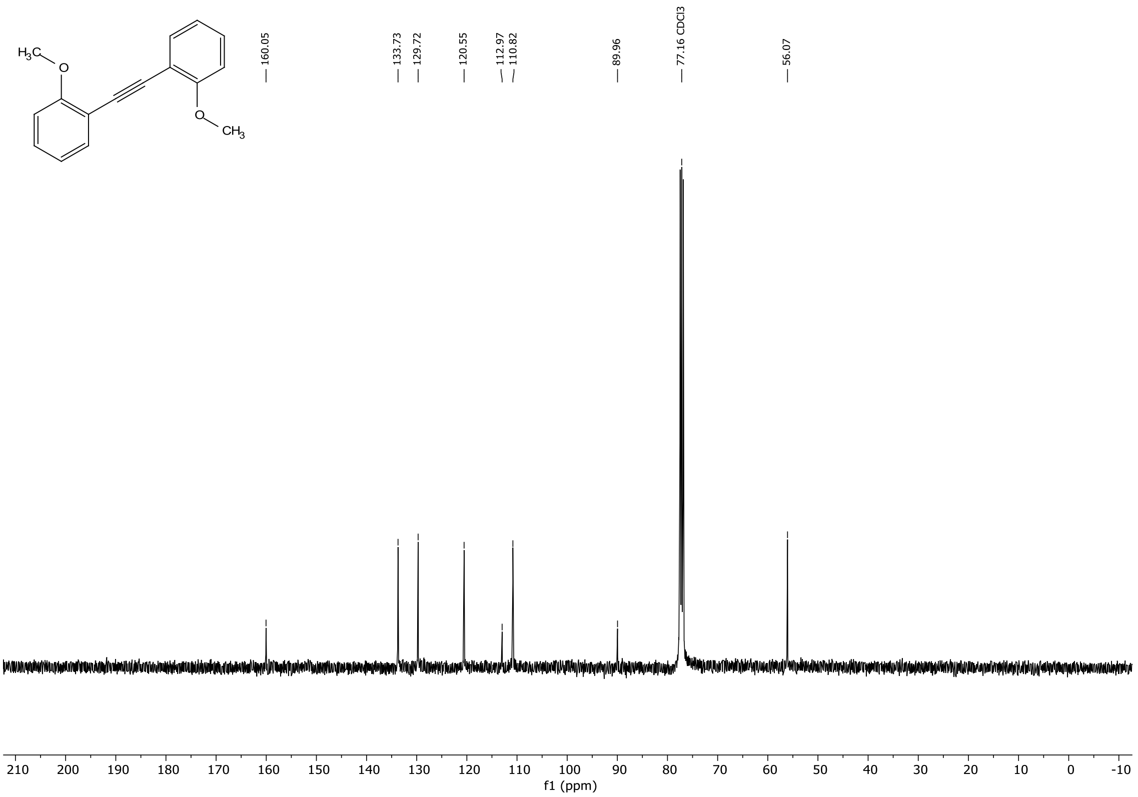

^{1}H NMR spectrum (400 MHz, $CDCl_3$) of **2j**.

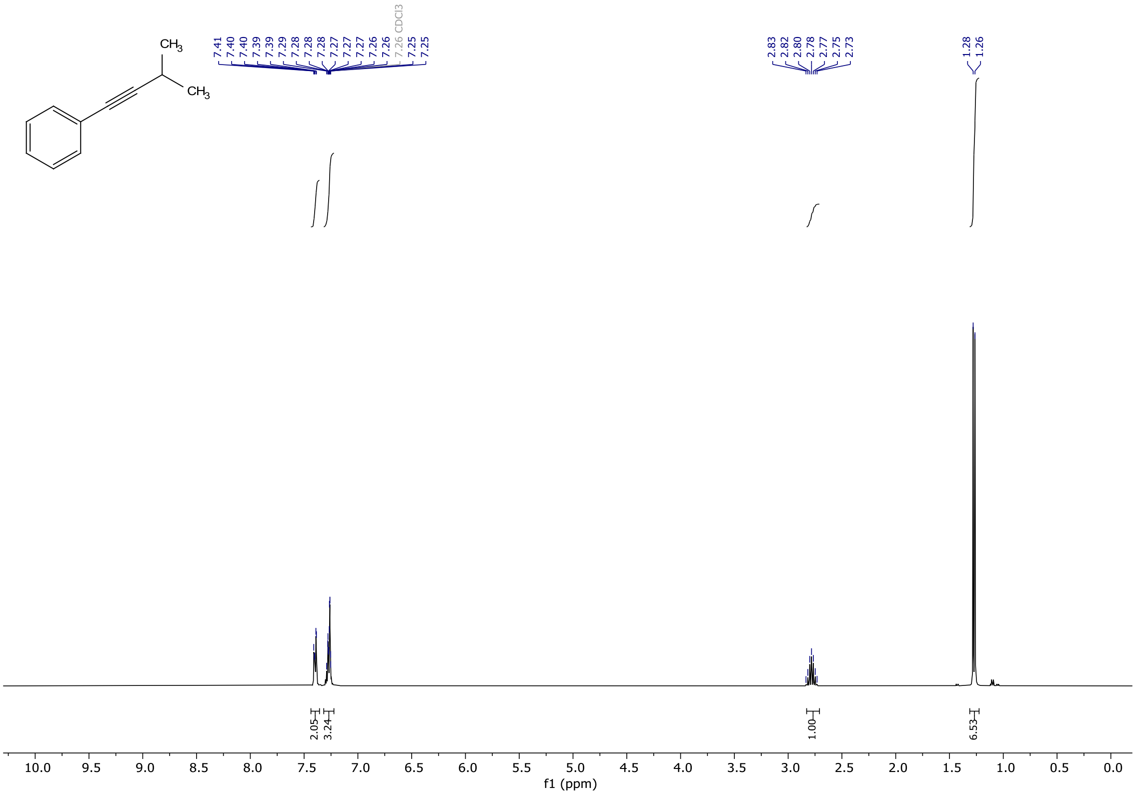

^{13}C NMR spectrum (101 MHz, $CDCl_3$) of **2j**.

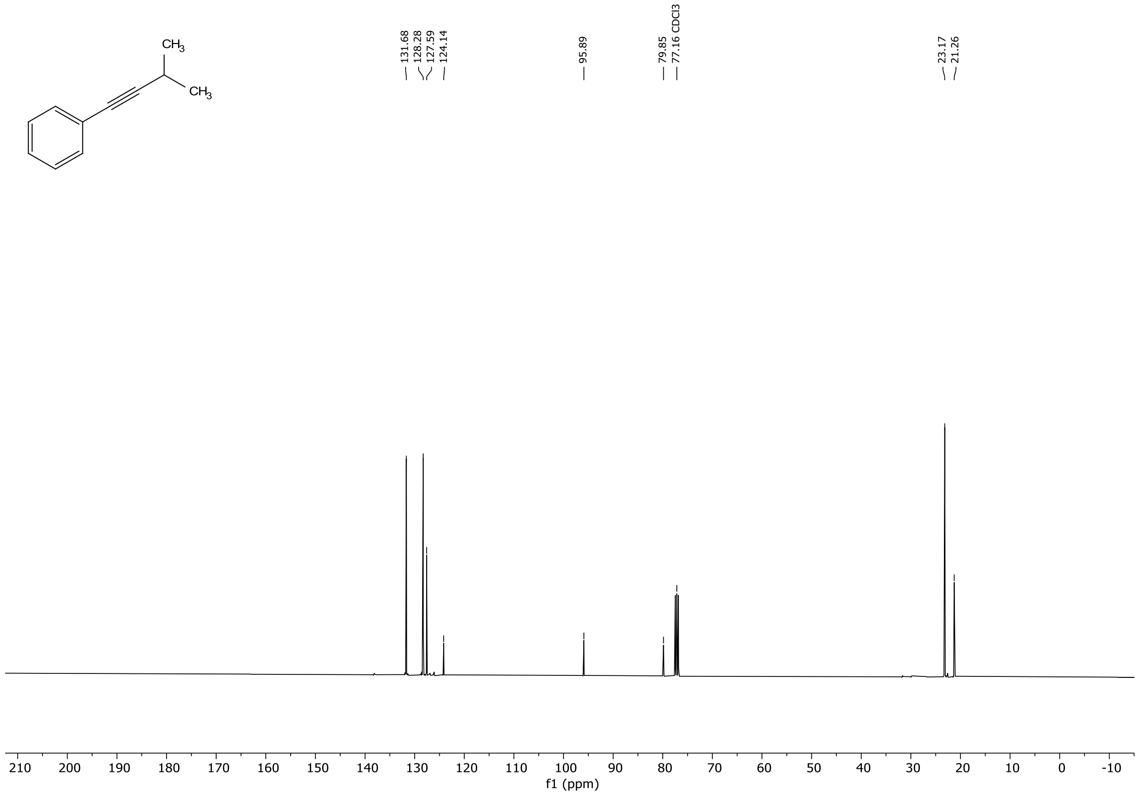

9. References

(1) Izutsu, K. *Electrochemistry in Nonaqueous Solutions*; Wiley, 2002. DOI: 10.1002/3527600655.

(2) Compton, R. G.; Banks, C. E. *Understanding voltammetry / Richard G Compton, Craig E Banks*, Fourth edition; World Scientific, 2025.

(3) Elgrishi, N.; Rountree, K. J.; McCarthy, B. D.; Rountree, E. S.; Eisenhart, T. T.; Dempsey, J. L. A Practical Beginner's Guide to Cyclic Voltammetry. *J. Chem. Educ.* **2018**, *95*, 197–206.

(4) Gütz, C.; Klöckner, B.; Waldvogel, S. R. Electrochemical Screening for Electroorganic Synthesis. *Org. Process Res. Dev.* **2016**, *20*, 26–32.

(5) Breitschaft, F. A.; Saak, A. L.; Krumbiegel, C.; Bartolomeu, A. d. A.; Weyhermüller, T.; Waldvogel, S. R. Multicomponent Electrosynthesis of Enaminyl Sulfonates Starting from Alkylamines, SO2, and Alcohols. *Org. Lett.* **2025**, *27*, 1210–1215.

(6) Weissman, S. A.; Anderson, N. G. Design of Experiments (DoE) and Process Optimization. A Review of Recent Publications. *Org. Process Res. Dev.* **2015**, *19*, 1605–1633.

(7) Hielscher, M. M.; Gleede, B.; Waldvogel, S. R. Get into flow: Design of experiments as a key technique in the optimization of anodic dehydrogenative C,C cross-coupling reaction of phenols in flow electrolyzers. *Electrochim. Acta* **2021**, *368*, 137420.

(8) Rüedi, G.; Oberli, M. A.; Nagel, M.; Weymuth, C.; Hansen, H.-J. A Practical and User-Friendly Method for the Selenium-Free One-Step Preparation of 1,2-Diketones and their Monoxime Analogs. *Synlett* **2004**, 2315–2318.

(9) Witham, C. A.; Mauleón, P.; Shapiro, N. D.; Sherry, B. D.; Toste, F. D. Gold(I)-catalyzed oxidative rearrangements. *J. Am. Chem. Soc.* **2007**, *129*, 5838–5839.

(10) Zhu, M.; Huang, S.; Chen, M.; Li, Y.; Zhong, M. A novel Schiff's base conjugate with multicolor changes in multiple states and its multipurpose applications for selective detection of Cu2+ in aggregated state and on silica gel strip. *Opt. Mater.* **2022**, *127*, 112288.

(11) Park, K.; Bae, G.; Moon, J.; Choe, J.; Song, K. H.; Lee, S. Synthesis of symmetrical and unsymmetrical diarylalkynes from propiolic acid using palladium-catalyzed decarboxylative coupling. *The Journal of organic chemistry* **2010**, *75*, 6244–6251.

(12) Luo, L.; Resch, D.; Wilhelm, C.; Young, C. N.; Halada, G. P.; Gambino, R. J.; Grey, C. P.; Goroff, N. S. Room-temperature carbonization of poly(diiododiacetylene) by reaction with Lewis bases. *J. Am. Chem. Soc.* **2011**, *133*, 19274–19277.

(13) Bheemireddy, S. R.; Ubaldo, P. C.; Rose, P. W.; Finke, A. D.; Zhuang, J.; Wang, L.; Plunkett, K. N. Stabilizing Pentacene By Cyclopentannulation. *Angew. Chem. Int. Ed.* **2015**, *54*, 15762–15766.

(14) Mio, M. J.; Kopel, L. C.; Braun, J. B.; Gadzikwa, T. L.; Hull, K. L.; Brisbois, R. G.; Markworth, C. J.; Grieco, P. A. One-pot synthesis of symmetrical and unsymmetrical bisarylethynes by a modification of the sonogashira coupling reaction. *Org. Lett.* **2002**, *4*, 3199–3202.

(15) Trosien, S.; Waldvogel, S. R. Synthesis of highly functionalized 9,10-phenanthrenequinones by oxidative coupling using MoCl5. *Org. Lett.* **2012**, *14*, 2976–2979.

(16) Munteanu, C.; Frantz, D. E. Palladium-Catalyzed Synthesis of Alkynes via a Tandem Decarboxylation/Elimination of (E)-Enol Triflates. *Org. Lett.* **2016**, *18*, 3937–3939.

(17) Liu, X.; Liu, R.; Dai, J.; Cheng, X.; Li, G. Application of Hantzsch Ester and Meyer Nitrile in Radical Alkynylation Reactions. *Org. Lett.* **2018**, *20*, 6906–6909.

(18) Wiebe, A.; Gieshoff, T.; Möhle, S.; Rodrigo, E.; Zirbes, M.; Waldvogel, S. R. Electrifying Organic Synthesis. *Angew. Chem. Int. Ed.* **2018**, *57*, 5594–5619.

(19) Pollok, D.; Waldvogel, S. R. Electro-organic synthesis - a 21st century technique. *Chem. Sci.* **2020**, *11*, 12386–12400.

(20) Cembellín, S.; Batanero, B. Organic Electrosynthesis Towards Sustainability: Fundamentals and Greener Methodologies. *Chem. Rec.* **2021**, *21*, 2453–2471.
